# High fat diet (HFD) induced hepatic lipogenic metabolism and lipotoxicity via Parkin-dependent mitophagy and Errα signal of *Pelteobagrus fulvidraco*

**DOI:** 10.1186/s40104-025-01200-1

**Published:** 2025-05-21

**Authors:** Angen Yu, Zhiwei Hao, Xiaolei Wei, Xiaoying Tan, Ester Zito, Hua Zheng, Zhi Luo

**Affiliations:** 1 Hubei Hongshan Laboratory, Fishery College, Huazhong Agricultural University, Wuhan, 430070 China; 2 Istituto Di Ricerche Farmacologiche Mario Negri IRCCS, Milan, 20156 Italy; 3 Department of Biomolecular Sciences, University of Urbino Carlo Bo, Urbino, 61029 Italy; 4 Laboratory for Marine Fisheries Science and Food Production Processes, Qingdao Marine Science and Technology Centre, Qingdao, 266237 China

**Keywords:** Errα, Hepatocytes, High fat diet, Lipid accumulation, Mitophagy, Parkin

## Abstract

**Background:**

Mitophagy is an essential cellular autophagic process which maintains mitochondrial homeostasis, but its role in high fat diet (HFD)-induced lipid accumulation is unclear in the yellow catfish. Thus, this study aimed to elucidate mechanism of mitochondria mediating HFD-induced hepatic fat accumulation.

**Results:**

In the present study, yellow catfish were fed three diets with dietary fat at 6.31% (low fat; LFD, control), 12.03% (middle fat; MFD) and 15.32% (high fat; HFD), respectively, for 8 weeks. High dietary fat addition raised hepatic lipid accumulation, and declined mRNA and protein levels of Parkin-dependent mitophagy, down-regulated the Parkin protein expression and the estrogen-related receptor alpha (Errα) ubiquitination, and induced Errα protein levels; fatty acid (FA) incubation reduced Parkin-dependent mitophagy, inhibited Errα ubiquitination and increased Errα protein expression, and raised TG accumulation. Furthermore, yellow catfish hepatocytes were isolated and cultured. Nicotinamide mononucleotide, *N*-acetyl-L-cysteine, Parkin and *errα* siRNA knockdown were used under FA incubation, respectively. Parkin downregulation mediated FA incubation-induced TG accumulation and mitoautophagic inhibition; Parkin ubiquitinated Errα, and K63 was an important ubiquitination site for deubiquitinating Parkin activity; Errα targets *fas*, *acca* and *pparγ* genes, whose activation contributed to FA-induced lipogenesis and lipid accumulation. Thus, high fat diet (HFD) and FA incubation inhibited Parkin activity, suppressed mitophagy and activated Errα pathway, and induced hepatic lipogenic metabolism and lipotoxicity.

**Conclusions:**

Overall, our study provided new targets against HFD-induced hepatic lipid accumulation and non-alcoholic fatty liver disease in the vertebrates.

**Supplementary Information:**

The online version contains supplementary material available at 10.1186/s40104-025-01200-1.

## Background

Lipids are essential for maintaining cellular energy homeostasis, and serve a lot of physiological functions in the vertebrates [1, 2]. However, high lipid intake usually induces excessive lipid accumulation with the vertebrates, leading to non-alcoholic fatty liver disease (NAFLD). The NAFLD is characterized by hepatic steatosis that is easily developed into non-alcoholic steatohepatitis (NASH), liver fibrosis and cancer [2, 3]. Notably, hepatic lipid accumulation is regulated by a complex network, including fatty acid transport, lipogenesis, and autophagy [3, 4]. Mitochondria are highly dynamic organelles and function in multiple metabolic processes, including nutrient metabolism, reactive oxygen species (ROS) generation, and dysfunctional mitochondria has been involved in the pathogenesis of NAFLD [5, 6]. Dysfunctional mitochondria can be discarded via mitophagy. In addition, the homeostasis of mitochondria can be maintained via the process of mitochondrial fission/fusion [6]. Thus, protecting mitochondria from lipotoxicity has emerged as a potential strategy for NAFLD treatment.

Mitophagy is an essential cellular autophagic process that selectively eliminates superfluous and damaged mitochondria [7, 8]. During the mitophagy, the autophagosome recognizes the damaged mitochondria in ubiquitin dependent or independent mechanisms through microtubule-associated protein 1 light chain 3 (LC3) adapters. E3 ubiquitin-protein ligase Parkin and PTEN-induced putative kinase 1 (PINK1) are known to function in ubiquitin-dependent mitophagy, while BCL-2 19-kDa interacting protein 3 (BNIP3), BCL2 like 13 (BCL2L13), FUN14 domain containing 1 (FUNDC1) and prohibitin 2 (PHB2) contribute to ubiquitin-independent mitophagy [8]. Studies suggested that NAFLD significantly inhibited mitophagy and defective mitophagy mediated the pathogenesis and progress of the fatty liver disease [9–11]. Furthermore, mitophagy sustained mitochondrial energy metabolism, attenuated high lipid-induced oxidative stress, thereby inhibiting the progression of NAFLD [12]. However, how HFD-induced lipid accumulation regulated mitophagy was largely unknown.

Parkin, an E3-ubiquitin ligase, is important in initiating mitophagy and performing quality control of mitochondria [13, 14]. It has been reported that the exercise increases Parkin protein levels to maintain mitochondrial quality control in NAFLD [15]. Meanwhile, the Parkin-dependent mitophagy can sustain mitochondrial function to alleviate HFD-induced NAFLD [16]. Moreover, other studies reveal that Parkin is a lipid-responsive regulator and modulates the CD36 ubiquitylation [17, 18]. Overall, these studies suggest that Parkin regulates multiple functions that contribute to lipid metabolism. However, the precise roles of Parkin-dependent mitophagy on NAFLD requires further investigation.

Estrogen-related receptor alpha (ERRα), the member of the orphan nuclear receptor, plays important roles in mitochondrial function and metabolism [19]. Previous report showed that ERRα-knockout mice was resistant to obesity induced by high fat intake [20, 21]. ERRα also plays an important role in controlling intestinal fat absorption and white adipose tissue (WAT) fat storage [21]. Furthermore, administration of ERRα inverse agonist reduced the circulating free fatty acid and triglyceride (TG) levels in obesity and diabetes rat models [22]. Interestingly, studies reported that Parkin bound to ERRα and increased their ubiquitination and degradation [23–25]. Therefore, since Parkin and Errα are key regulators for mitochondrial homeostasis and lipid metabolism, we speculated that Errα protein is a potential target for high fat-induced lipid accumulation, and Parkin-Errα axis is likely to be a potentially important pathway for regulation.

Teleost fish have approximately 30,000 species and are the largest group among vertebrates. During the evolution, fish were thought to undergo a specific fish-specific genome duplication (FSGD) [26]. Based on available whole-genome sequences, the FSGD has been reported in yellow catfish *Pelteobagrus fulvidraco*, a widely distributed freshwater fish in Asia [27]. After FSGD, new genes evolved, which potentially have new functions and regulatory mechanism. Therefore, we attempted to elucidate new regulatory mechanisms of lipid metabolism by using yellow catfish as a model. Our objectives were to determine: 1) whether and how mitochondria mediated the process of HFD-induced lipid accumulation; 2) whether and how Parkin-dependent mitophagy was involved in HFD-induced lipid accumulation; and 3) whether and how Errα signal mediated HFD-induced lipid accumulation.

## Materials and methods

### Exp. 1: in vivo study: animal feeding experiment

The feeding experiment was similar to our previous study with minor modification [28]. Briefly, 270 yellow catfish (body weight: 2.33 ± 0.03 g, one-month-old) were randomly stocked in 9 tanks, 30 fish per tank. Each experimental diet was allocated to three tanks. Three diets were formulated with dietary fat at 6.31% (low-fat diet; LFD, the control), 12.03% (middle-fat diet; MFD), and 15.32% (high-fat diet; HFD) (Supplemental Table S1). Yellow catfish were fed every day at 8:00 and 16:00, respectively, to satiation for 8 weeks. During the feeding period, the water temperature was 27.8 ± 0.5 °C, the dissolved oxygen was 6.01 ± 0.17 mg/L, the pH was 7.81 ± 0.22 and the NH_4_-N was 0.10 ± 0.04 mg/L.

At the finish of the trial, yellow catfish were weighed to determine the growth performance. The liver samples were randomly collected from four fish in each tank and utilized for histological, histochemical, and ultrastructural structure. Additionally, liver samples from another four fish in each tank were obtained for the assessment of biochemical parameters, such as enzyme activities. Moreover, the liver of another four fish from each tank were sampled for the analysis of gene and protein expression. Prior the analysis, all samples were rapidly frozen in liquid nitrogen and subsequently stored in an −80 °C freezer.

### Exp. 2: in vitro study

#### Part 1. Yellow catfish hepatocytes culture and treatments

Yellow catfish hepatocytes were isolated based on the protocols in our recent publication [29], and the details were shown in Supplemental Text S1. To investigate the mechanism through which dietary fat influenced mitophagy and lipid metabolism in hepatocytes, we performed several in vitro experiments. Firstly, we designed two treatments, such as the control (without extra FA addition) and FA group (0.4 mmol/L) with palmitic acid (PA) and oleic acid (OA) at a ratio of 1:1, according to previous report [30]. NMN (nicotinamide mononucleotide) was used as the mitophagy activator to investigate the mechanism of mitophagy in FA-induced variations of lipid metabolism. For small interfering RNA (siRNA) experiments, *parkin* and *errα* knockdown via specific siRNA were performed in yellow catfish hepatocytes, as described in our publication [31]. The siRNA knockdown sequences were given in Supplemental Table S3.

#### Part 2. HEK293 T cell culture, treatment and construction of plasmids

To explore the underlying mechanisms connecting mitophagy and lipid metabolism after FA incubation, we constructed several vectors based on our published protocols [32], and the details was shown in Supplemental Text S2. The primers for these plasmids are listed in Supplemental Table S4. The HEK293 T (human embryonic kidney) cells were used to explore gene function because of their high transfection efficiency [31]. Their primer sequences are given in Supplemental Table S4. The plasmids were transfected into the HEK293 T cells via the Lipofectamine 2000 (11668019; Invitrogen).

### Sample analysis

#### Histological and ultrastructural analysis

The protocols for hematoxylin and eosin (H&E), Oil Red O, and Sirius Red staining were shown in details in Supplemental Text S3. The mitochondrial morphology in liver and hepatocytes were detected via the FEI Tecnai G^2^ 20 TWIN transmission electronic microscope (TEM, Leica Microsystems, Wetzlar, Germany), according to the manufacturer’s protocols.

#### Analysis of nutrient and fatty acid composition

The content of moisture, crude protein, lipid, and ash in the feed were determined based on the Standard methods [33], and the detailed protocols were shown in Supplemental Text S4. The fatty acid composition of the feed was analyzed based on the previous study [34], and the detailed protocols were shown in Supplemental Text S4.

#### Cell viability, total triglyceride (TG) and ATP contents measurement

Cell viability was analyzed via the commercial kit (C0037, Beyotime, Beijing, China). TG contents were measured by specific assay kits (Nanjing Jiancheng Bioengineering Institute, Nanjing, China). The ATP levels were determined via an ATP Assay kit (S0026A, Beyotime). Protein concentrations were analyzed by the BCA protein assay kit (P0012S, Beyotime).

#### RNA isolation and real-time quantitative PCR (qPCR) analysis

For liver tissues and primary hepatocytes, RNA was isolated by using TRIzol reagent (Sigma-Aldrich). The detailed protocol was shown in Supplemental Text S5. The primer sequences were listed in Supplemental Table S5. Seven housekeeping genes [*β-actin,* ubiquitin-conjugating enzyme (*ubce*)*,* glyceraldehyde-3-phosphate dehydrogenase (*gapdh*)*,* 18S ribosomal RNA (*18s rRNA*)*,* tubulin-A (*tuba*)*,* beta2-microglobulin (*b2m*)*,* and ribosomal protein L7 (*rpl7*)] were measured, and the optimal combination of two genes were determined via the online tool geNorm. The fold change in mRNA expression was quantified via the 2^−∆∆CT^ method [35].

#### Western blot, immunoprecipitation (IP) and immunofluorescence assays

Western blot assay (WB) was performed to determine the protein expression, and the detailed protocol was shown in Supplemental Text S6. The availability of antibodies has been verified during the preliminary experiment and studies [29, 31, 32]. The protein bands were visualized by an Odyssey infrared fluorescent imaging system (Li-Cor Biosciences) and quantified using Image 2.0.0 version software (USA). IP assays and immunofluorescence staining were used to analyze the protein interactions, and the detailed protocols were shown in Supplementary Text S6. 

#### Mitochondrial isolation and DNA depletion

Mitochondria were isolated from the liver and hepatocytes via the differential centrifugation technique, as described previously [35]. Genomic DNA was extracted using DNAzol (Thermo Fisher Scientific). qPCR was performed on mitochondrial *atp8* gene and normalized to *eeflα* gene. The primer sequences are listed in Supplemental Table S5.

#### Luciferase activity measurement and electrophoretic mobility shift assay

Luciferase activity was measured via the Dual Luciferase Reporter Assay System (Promega, Minneapolis, USA), and the activity was represented as the ratio of the Firefly luciferase to the Renilla luciferase. The specific primers were detailed in Supplemental Table S6. The EMSA was utilized to identify the direct binding sites of promoter regions, as described in Yu et al. [31], and the specific primers were given in Supplemental Table S7.

### Statistical analysis

The statistical analyses were performed via the software SPSS 22.0 (IBM, USA) and presented as mean ± SEM unless otherwise noted in the figure legends. Before processing percentage data, we first used an arcsine transformation. Then, we used the Kolmogorov-Smirnov test to analyze the distribution normality among the data, and tested the homogeneity of variances among treatments via the Barlett’s test. One-way ANOVA with Duncan’s multiple range test was utilized to analyze the experimental data in our in vivo study (3 treatments). The data between two treatments in the in vitro study was conducted using the Student’s *t*-test. The Student's *t*-test was also used to analyze the results of the effects of the siRNA experiments in our in vitro study. All figures were drawn using Graph—Pad Prism 8.0 software (USA). *P*-values < 0.05 was considered the threshold for statistical significance.

## Results

### Exp. 1: in vivo study

#### Fatty acid composition of feeds, growth performance and feed utilization

The fatty acid composition of feeds is presented in Supplemental Table S8. As expect, the contents of saturated fatty acids (SFA), mono-unsaturated fatty acids (MUFA), n-3 poly-unsaturated fatty acids (n-3 PUFA) and n-6 poly-unsaturated fatty acids (n-6 PUFA) were increased with the lipid content. Notably, the percentage of SFA was significantly increased with lipid contents, as shown in Supplemental Table S9. After 8 weeks, the survival was not different across the three groups. Compared with the LFD group, specific growth rate (SGR), weight gain (WG) and condition factor (CF) increased with dietary fat content. Compared with LFD and MFD, the HFD increased feed intake (FI). Hepatosomatic index (HSI) was higher in MFD and HFD than the LFD. Feed efficiency (FE) was not significantly different among three groups (Supplemental Table S2). Taken together, high dietary fat addition promoted a significant growth of yellow catfish.

#### High dietary fat addition up-regulated lipogenesis and induced hepatic lipid accumulation

**Fig. 1:**
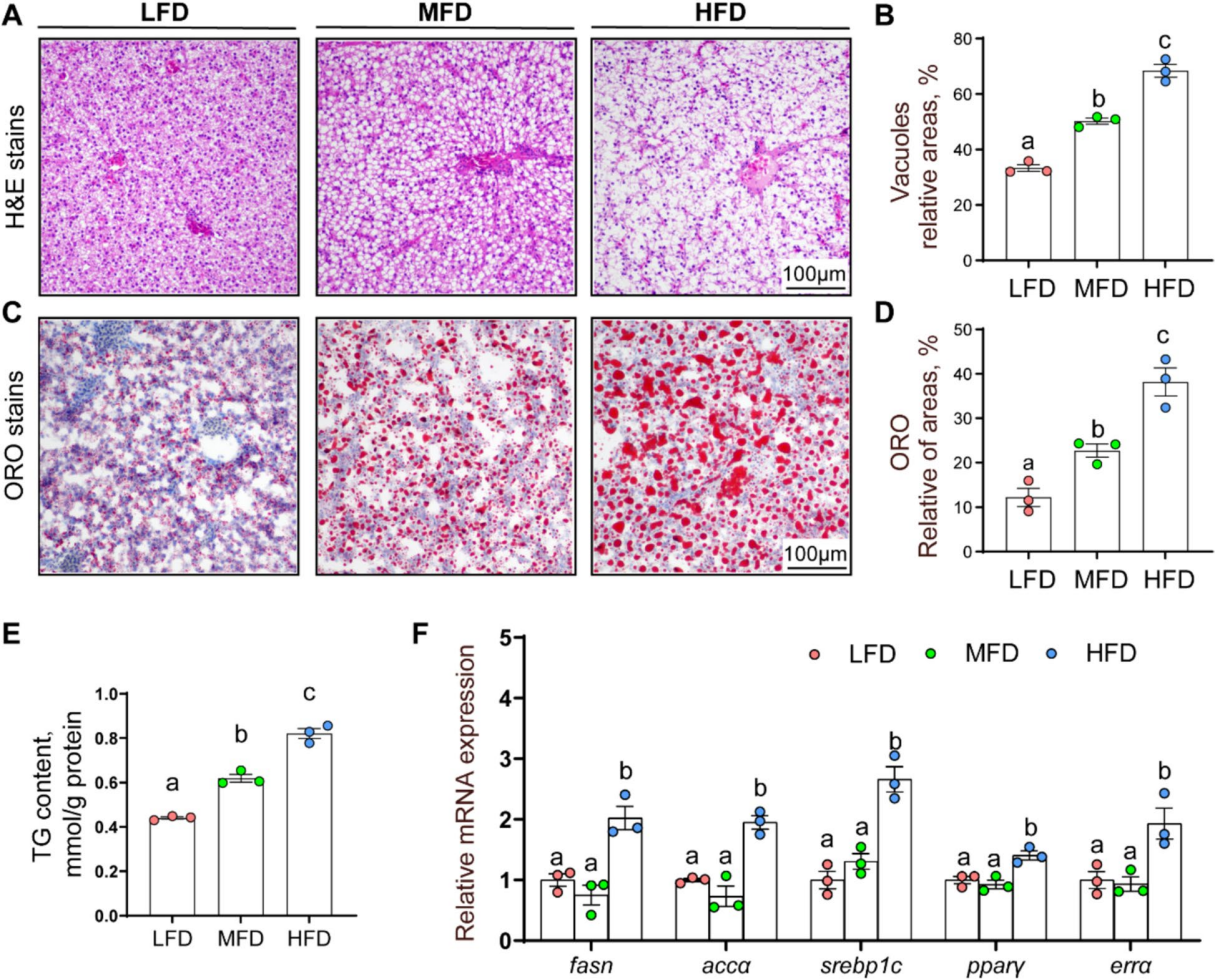
Dietary fat addition induced hepatic lipid accumulation in the yellow catfish. **A** and **B** Relative areas for H&E staining. scale bars, 100 µm. **C** and **D** Relative areas for Oil-red O staining. scale bars, 100 µm. **E** TG contents. **F** mRNA expression of lipid metabolism related genes. Values are means ± SEM (*n* = 3 replicate tanks, 6 fish were sampled from each tank). Labeled means without a common letter differ, *P* < 0.05 (one‑way ANOVA, Duncan post hoc test)

Compared to LFD group, the MFD and HFD groups raised the amount of hepatic lipid droplets (LDs) (Fig. 1A and B), and increased the Oil Red O–relative areas (Fig. 1C and D) and hepatic TG content (Fig. 1E). Compared to LFD and MFD groups, the mRNA abundance of lipogenic genes [fatty acid synthase (*fas*), acetyl CoA carboxylase (*accα*), sterol regulatory element binding protein 1c (*srebp1c*), peroxisome proliferator activated receptor γ (*ppar-γ*), and estrogen related receptor alpha (*errα*)] were up-regulated in HFD group (Fig. 1F), indicating that high dietary fat upregulated lipogenic metabolism and accordingly lipid accumulation.

#### High dietary fat addition facilitated hepatic mitochondrial fragmentation and inhibited mitophagy

**Fig. 2:**
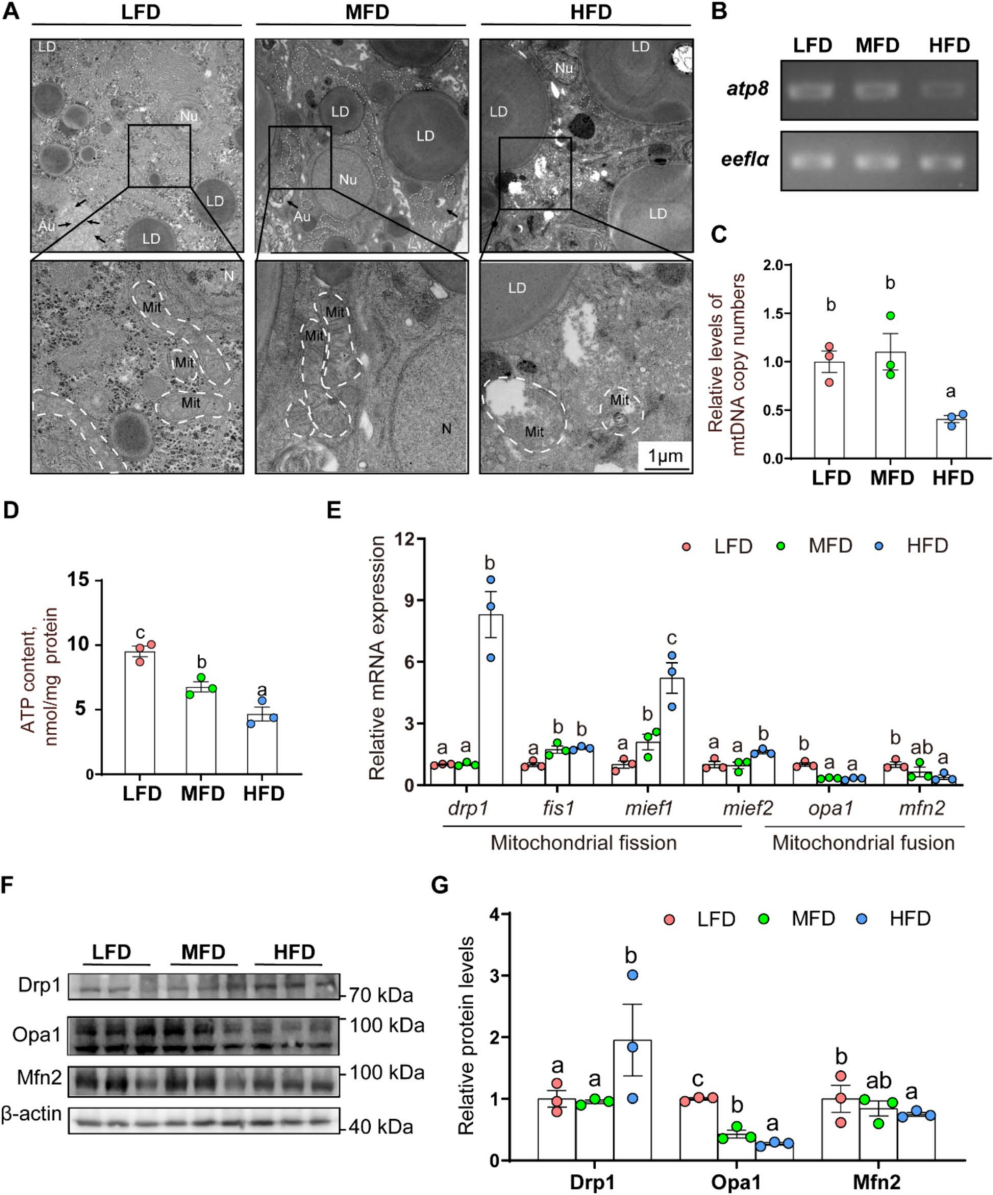
Dietary fat addition induced hepatic mitochondrial fragmentation and disrupted the mitochondrial function. **A** liver ultrastructure; **B** *atp8* mRNA expression; **C** Related levels of mt-DNA copy numbers; **D** ATP content; **E** Relative mRNA expression of mitochondrial fission and fusion related genes; **F** and **G** Relative protein concentrations of mitochondrial fission and fusion. Values are means ± SEM (*n* = 3 replicate tanks, 6 fish were sampled from each tank). Labeled means without a common letter differ, *P* < 0.05 (one‑way ANOVA, Duncan post hoc test)

Mitochondria are key intracellular energy-supplying organelle, and mitochondrial function is determined by their structural integrity [6]. Compared with LFD and MFD groups, TEM observation indicated that hepatic mitochondria from HFD group were swollen and damaged (Fig. 2A), and the HFD group significantly decreased the mitochondrial copy numbers and ATP content (Fig. 2B–D). Mitochondrial fission and fusion processes are important for maintaining mitochondrial dynamics [38]. In this study, HFD group raised the mRNA abundance of mitochondrial fission-related genes [fission, dynamin related protein 1 (*drp1*), mitochondrial 1 (*fis1*), mitochondrial elongation factor 1 (*mief1*) and 2 (*mief2*)], but downregulated the mRNA abundance of mitochondrial fusion-related genes [optic atrophy 1 (*opa1*) and mitofusin 2 (*mfn2*)] (Fig. 2E). Furthermore, compared to the LFD and MFD groups, HFD group increased Drp1 protein expression, and reduced the Opa1 and Mfn2 protein expression (Fig. 2F and G). Thus, high dietary fat addition facilitated hepatic mitochondrial fragmentation.

**Fig. 3:**
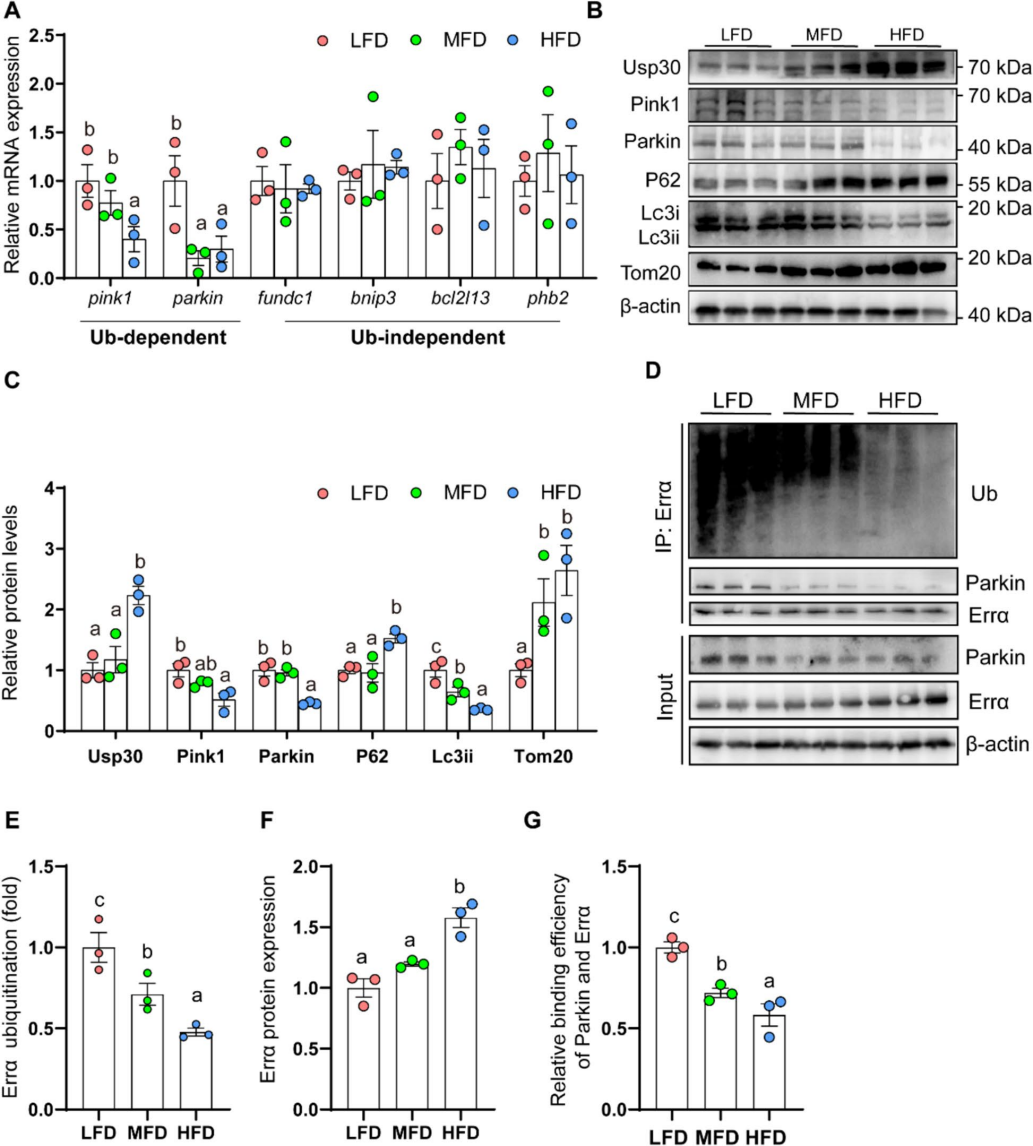
Dietary fat addition inhibited mitophagy and Errα ubiquitination. **A** Relative mRNA expression of mitophagy related genes; **B** and **C** Relative protein concentrations of mitophagy; **D** and **E** The Errα ubiquitination and interaction between Parkin and Errα; **D** and **F** Relative protein concentrations of Errα; **D** and **G** Relative binding efficiency of Parkin and Errα. Values are means ± SEM (*n* = 3 replicate tanks, 6 fish were sampled from each tank). Labeled means without a common letter differ, *P* < 0.05 (one‑way ANOVA, Duncan post hoc test)

To further explore the link between mitochondrial function and lipid metabolism, the key indicators for the mitophagy were analyzed. Compared to LFD group, HFD group significantly reduced the mRNA expression of Parkin-dependent mitophagic genes (*pink1* and *parkin*), but did not influence the mRNA abundance of Parkin-independent mitophagy-related genes (*fundc1*, *bnip3*, *bcl2l13* and *phb2*) (Fig. 3A), indicating that high dietary fat reduced the Parkin-dependent mitophagy. Furthermore, compared to LFD group, HFD group significantly reduced Pink1, Parkin and Lc3ii protein expression (*P* < 0.05) (Fig. 3B and C), but raised Usp30, P62 and Tom20 protein expression (*P* < 0.05), further indicating that HFD inhibited Parkin-dependent mitophagy. ERRα is a potential target protein of Parkin, and plays a major regulatory role in lipid accumulation [23, 25] Compared to LFD and MFD groups, HFD group inhibited the Errα ubiquitination and up-regulated the Errα protein level (Fig. 3D and F). Immunoprecipitation analysis indicated that the MFD and HFD groups inhibited the interaction between Parkin and Errα than LFD group (Fig. 3D and G). Thus, HFD inhibited mitophagy, reduced the interaction between Parkin and Errα, and inhibited the Errα ubiquitination degradation and up-regulated the Errα protein level.

### Exp. 2: in vitro study

#### FA incubation induced lipid accumulation, reduced Parkin expression and mitophagy, inhibited Errα ubiquitination and increased Errα protein expression in yellow catfish hepatocytes

**Fig. 4:**
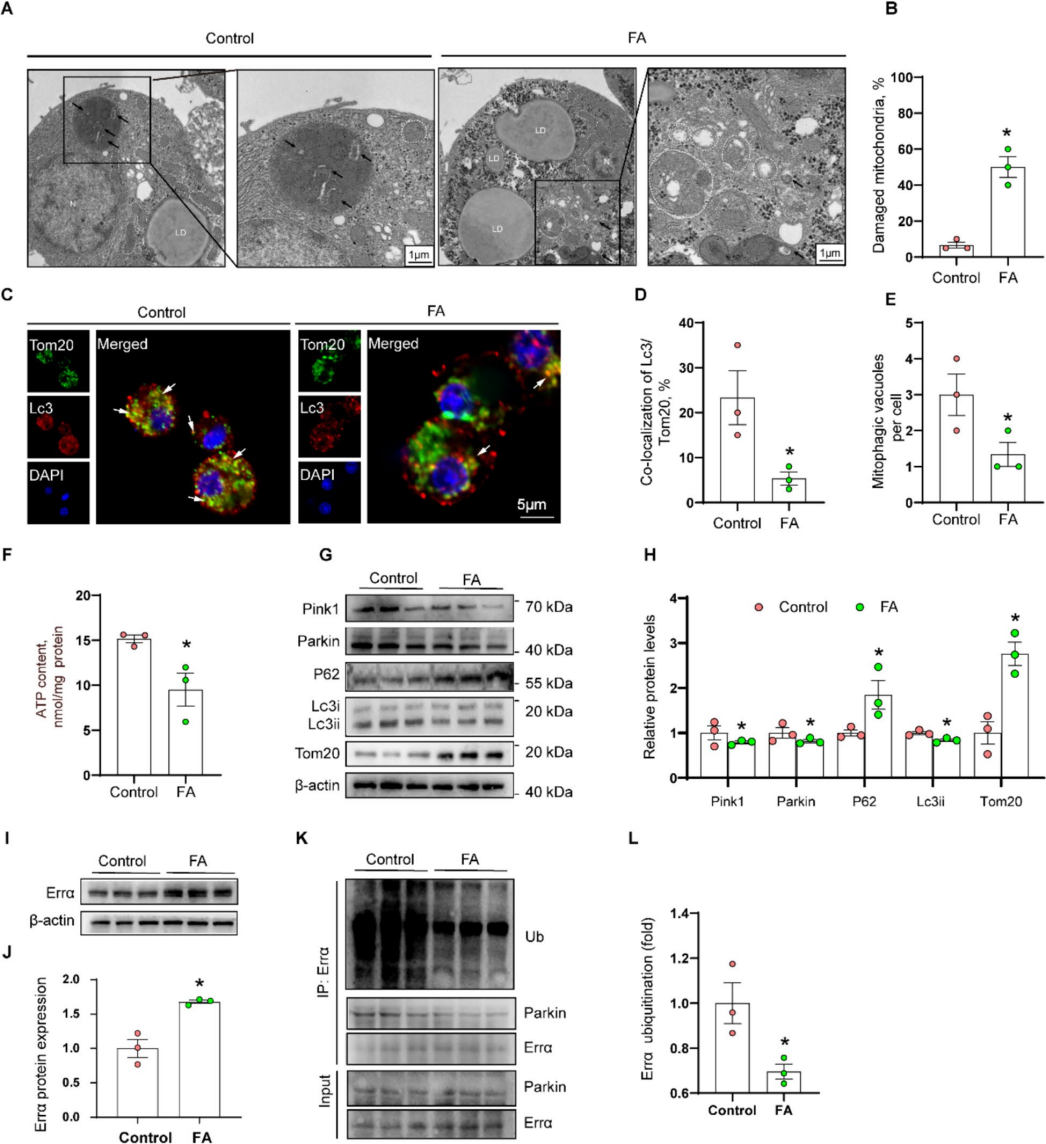
FA incubation raised lipid accumulation and reduced Parkin-dependent mitophagy and Errα ubiquitination in yellow catfish hepatocytes. **A** and **B** The ultrastructure (TEM) of hepatocytes; **C–E** Representative confocal microscopic image of hepatocytes stained with Lc3 and Tom20; **F** ATP content; **G** and **H** Relative protein concentrations of Parkin-dependent mitophagy; **I** and **J** Relative protein concentrations of Errα; **K** and **L** The ubiquitination of Errα under FA incubation. Values are means ± SEM (*n* = 3 represents three independent experiments, and three replicates were used for each independent biological experiments). Asterisks (*) indicate significant differences between two corresponding groups (*P* < 0.05), *P* value was calculated by Student's *t*-test

FA concentrations < 0.4 mmol/L did not affect cell viability (Supplemental Fig. S1). Hepatocyte TG content increased with FA concentration (Supplemental Fig. S2). Thus, the 0.4 mmol/L FA was used for the in vitro experiment because it induced TG accumulation but did not affect the cell viability. TEM observation showed that the mitochondria were swollen and damaged under FA incubation, and the mitophagosomes was decreased under FA incubation **(**Fig. 4A and B). The immunofluorescence further confirmed that FA incubation inhibited mitophagy (Fig. 4C–E). Compared with the control, FA incubation significantly downregulated ATP content (Fig. 4F), reduced Pink1, Parkin and Lc3ii protein expression, and upregulated the P62 and Tom20 protein expression (Fig. 4G and H), indicating FA incubation inhibited Parkin expression and mitophagy in yellow catfish hepatocytes. Thus, FA incubation raised lipid accumulation, reduced Parkin expression and mitophagy.

Next, compared to the control, FA incubation significantly increased Errα protein expression (Fig. 4I and J). Meanwhile, immunoprecipitation results revealed that FA incubation reduced the ubiquitination of Errα (Fig. 4K and L), Thus, similar to the in vivo study, FA incubation inhibited Errα ubiquitination and increased Errα protein expression in yellow catfish hepatocytes.

#### The inhibition of mitophagy contributed to FA-induced lipid accumulation

**Fig. 5:**
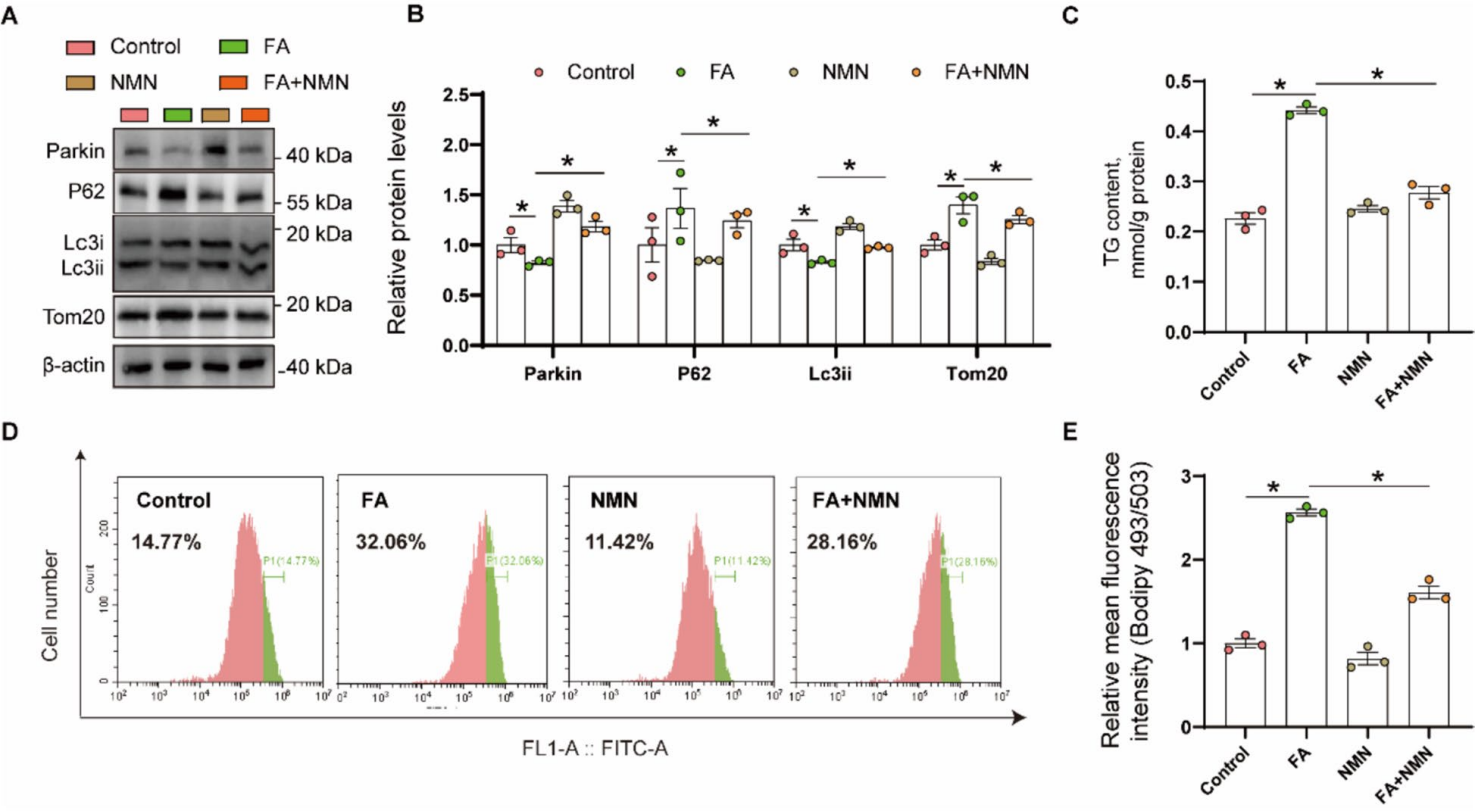
Mitophagy inhibition contributed FA incubation-induced lipid accumulation of yellow catfish hepatocytes. **A** and **B** Relative protein concentrations of Parkin-dependent mitophagy; **C** TG content; **D** Presence of LDs stained with Bodipy 493/503 was demonstrated by flow cytometry; **E** Lipid content was quantified by flow cytometric analysis of FL1 (green) mean fluorescence intensity with BODIPY 493/503 staining. Values are means ± SEM (*n* = 3 independent biological experiments, and three replicates were used for each independent experiment). Asterisks (*) represent significant differences at *P* < 0.05 between the two groups, as determined using Student's *t*-test

Lipid accumulation and lipotoxicity cause mitochondrial damage [6], but mitophagy prevents the accumulation of damaged mitochondria [7, 8]. In order to explore whether and how mitophagy mediated FA incubation-induced lipid accumulation, we chose nicotinamide mononucleotide (NMN) that can activate the Parkin-dependent mitophagy pathway [30], for the following study. NMN concentrations ≤ 0.8 mmol/L did not adversely affect cell viability (Supplemental Fig. S3). As expected, FA and NMN co-treatment interacted to affect the protein expression of mitophagic genes (Parkin, Lc3ii, P62 and Tom20) (Fig. 5A and B). Compared with the control, FA incubation reduced Parkin and Lc3ii protein expression, and NMN treatment alleviated the FA-induced reduction of Parkin and Lc3ii protein expression. Compared to the control, FA incubation induced the P62 and Tom20 protein expression. The NMN treatment abrogated the FA-induced increment of P62 and Tom20. Taken together, NMN treatment alleviated the FA-induced inhibition of mitophagy in yellow catfish hepatocytes.

Next, we attempted to explore mechanism of mitophagy mediating FA-induced lipid accumulation. FA incubation and NMN treatment interacted to affect the TG contents in yellow catfish hepatocytes. Compared with control, FA incubation increased the content of TG, and NMN treatment reversed the trend (Fig. 5C). Meanwhile, FA incubation induced the amounts of lipid droplets and NMN treatment alleviated the amounts of lipid droplets induced by FA (Fig. 5D and E). Thus, the inhibition of mitophagy contributed to FA incubation-induced lipid accumulation.

#### FA-induced lipid accumulation and autophagic inhibition are Parkin-dependent

**Fig. 6:**
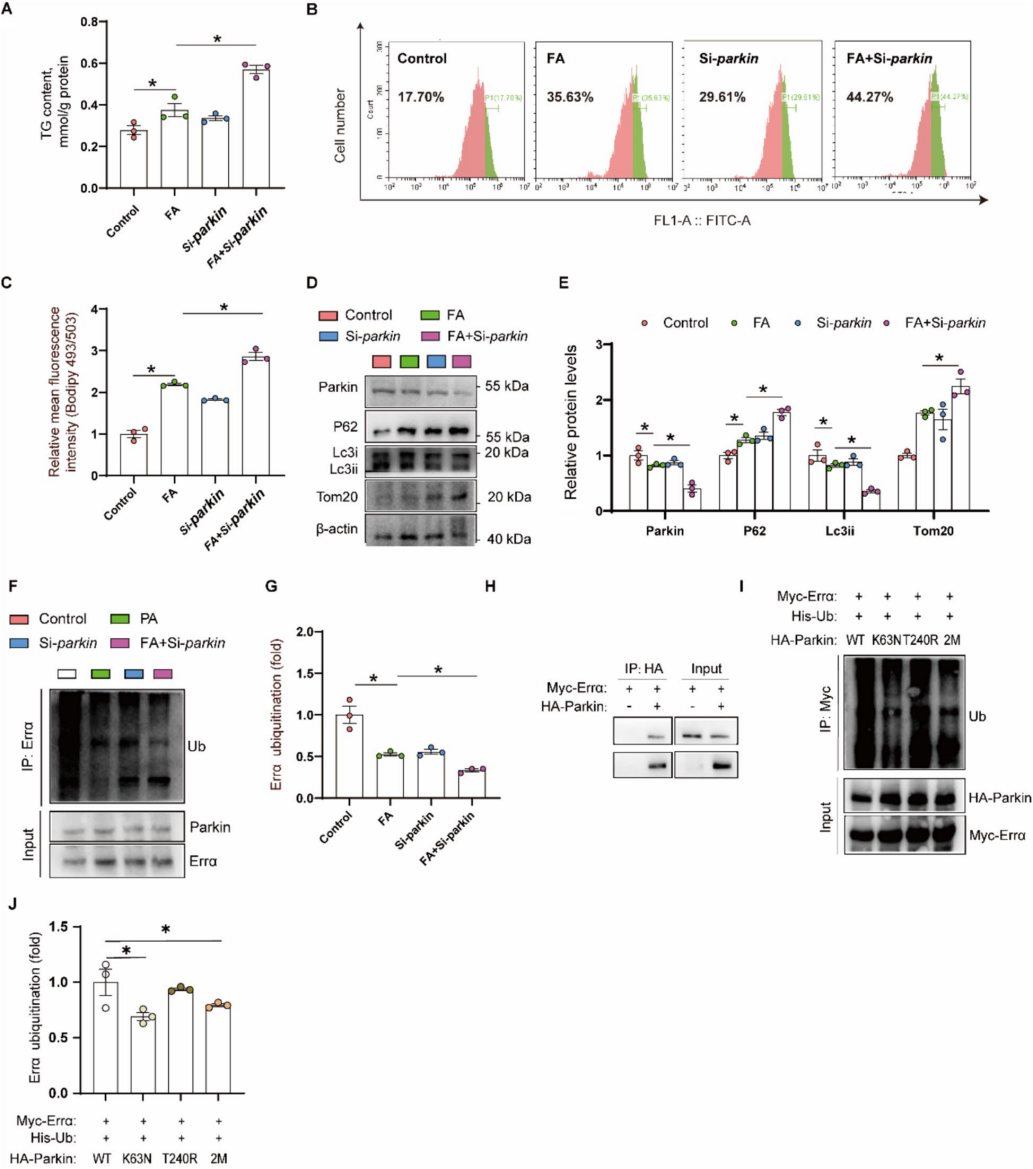
FA-induced lipid deposition, mitophagic inhibition and ERRα ubiquitination were Parkin-dependent. **A** TG content; **B** Presence of LDs stained with Bodipy 493/503 was demonstrated by flow cytometry; **C** Lipid content was quantified by flow cytometric analysis of FL1 (green) mean fluorescence intensity with BODIPY 493/503 staining; **D** and **E** Relative protein concentrations of Parkin-dependent mitophagy; **F** and **G** The Errα ubiquitination; **H** Interaction of Parkin with Errα, HA‑tag Parkin and Myc‑tag Errα were transfected into 293 T cells, and the interaction between Parkin with Errα was determined with IP and western blot; **I** and **J** The Errα de-ubiquitination. Values are means ± SEM (*n* = 3 independent biological experiments, and three replicates were used for each independent experiment). Asterisks (*) represent significant differences at *P*  < 0.05 between the two groups, as determined using Student's *t*-test

To elucidate mechanisms of Parkin in FA-induced variation of lipid accumulation and mitophagy, we employed small interfering RNA to knockdown the expression of *parkin*. The *parkin* siRNA-422 was selected for our in vitro studies due to its remarkable inhibitory efficiency on *parkin* mRNA expression (Supplemental Fig. S4A). FA treatment and *parkin*-siRNA experiment interacted to affect the TG contents in yellow catfish hepatocytes. Compared with the control group, FA incubation increased TG content, and *parkin*-knockdown exacerbated the FA-induced increase of TG content (Fig. 6A). Meanwhile, FA incubation induced the amounts of lipid droplets and *parkin*-knockdown alleviated the amounts of lipid droplets induced by FA (Fig. 6B and C). Thus, FA incubation-induced lipid accumulation was Parkin-dependent.

Next, we demonstrated influences of *parkin* knockdown on mitophagy in yellow catfish hepatocytes. FA incubation and *parkin* siRNA interacted to affect the protein expression of mitophagic genes (Parkin, Lc3ii, P62 and Tom20) (Fig. 6D and E). Compared to the control, FA incubation reduced Parkin and Lc3ii protein expression, and *parkin*-siRNA exacerbated the FA-induced reduction of Parkin and Lc3ii protein expression. FA incubation also induced the P62 and Lc3ii protein expression, and the *parkin*-siRNA exacerbated the FA-induced increment of P62 and Lc3ii protein expression. Thus, FA incubation-induced inhibition of mitophagy was Parkin-dependent.

#### Parkin was the potential E3-ubiquitin ligase of Errα, whose inhibition contributed to FA-induced reduction of the Errα ubiquitination

Next, we explored the mechanism of HFD and FA influencing the ubiquitination of Errα. We found that FA incubation and *parkin*-siRNA interacted to affect the ubiquitination of Errα in yellow catfish hepatocytes. Compared with the control, the Errα ubiquitination was reduced under FA incubation, and *parkin*-knockdown further exacerbated the reduction of Errα ubiquitination under FA incubation (Fig. 6F and G). The Parkin coprecipitated with Errα was also verified in the HEK293 T cells after HA-Parkin and Myc-Errα overexpression (Fig. 6H). Then, Myc‑tag Errα and HA‑tag Parkin or mutants were transfected into the 293 T cells. Compared to the WT, the single (K63 N) and double (K63 N and T240 N) mutants of Parkin reduced the ubiquitination of Errα, but the single (T240 N) mutant could not affect the Errα ubiquitination (Fig. 6I and J). Thus, Parkin was the potential E3-ubiquitin ligase of Errα, whose inhibition contributed to FA-induced reduction of the Errα ubiquitination, and K63 was an important site for deubiquitinating Parkin activity.

#### ERRα targets *fas*, *acca* and *pparγ* genes, whose activation contributed FA-induced lipid accumulation

**Fig. 7:**
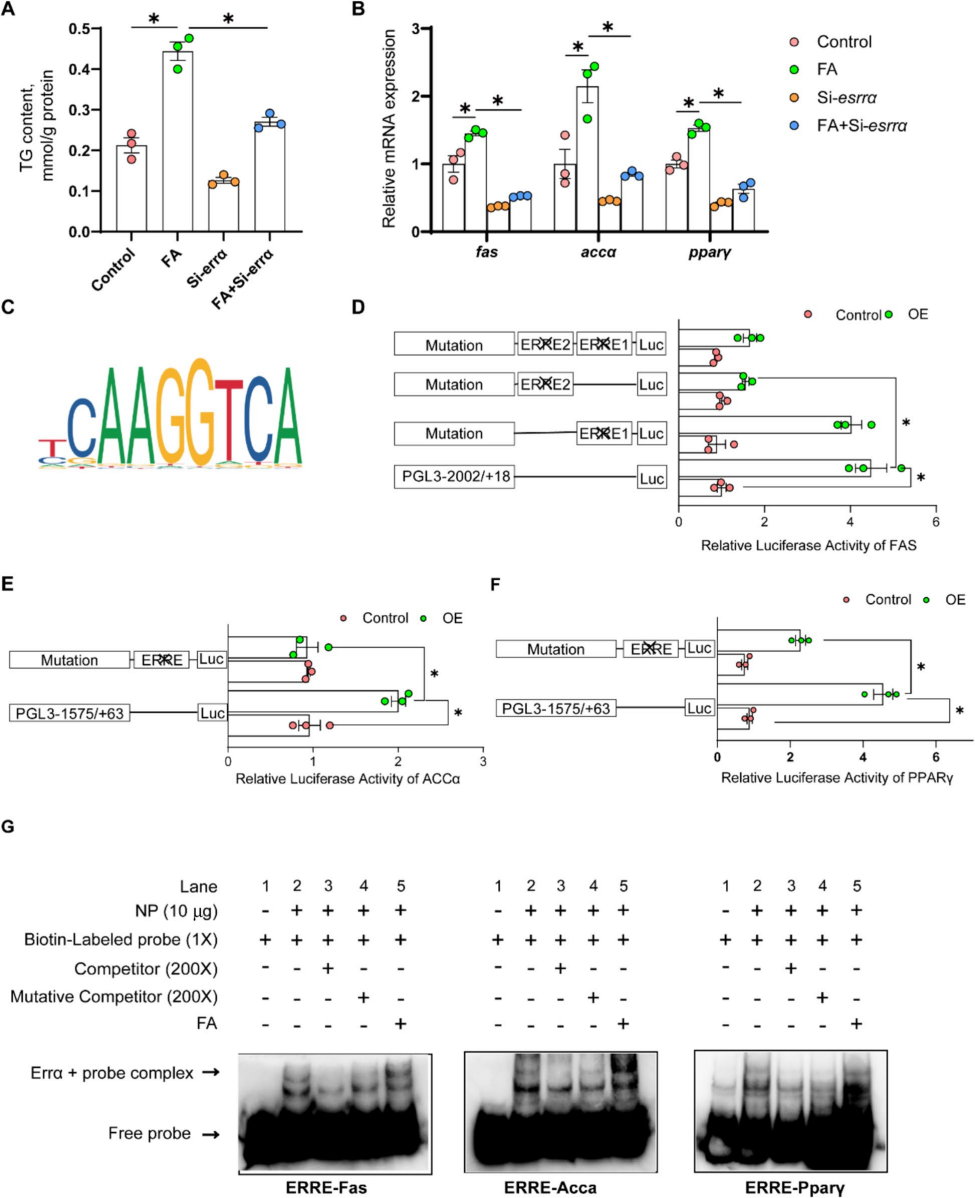
ERRα targeted *fas*, *acca* and *pparγ* genes, whose activation contributed to FA-induced lipid accumulation. **A** TG content; **B** mRNA levels of lipogenic genes; **C** Sequence logo of ERRα; **D** Relative luciferase activity of *fas* promoter; **E** Relative luciferase activity of *accα* promoter; **F** The relative luciferase activity of pparγ promoter; **G** EMSA of putative ERRα binding sequences (ERRE). Values are means ± SEM (*n* = 3 independent biological experiments, and three replicates were used for each independent experiment). Asterisks (*) represent significant differences at *P*  < 0.05 between the two groups, as determined using Student's *t*-test

Next, the siRNA experiment was performed to elucidate mechanism by which Errα mediated hepatic lipogenesis. The *errα* siRNA-796 was selected due to its remarkable inhibitory efficiency on *errα* mRNA expression (Supplemental Fig. S4B). FA and *errα*-siRNA interacted to affect the TG contents in yellow catfish hepatocytes. Compared to the control, the TG content was significantly induced under FA incubation, and *errα*-knockdown reversed the increase of TG content under FA incubation (Fig. 7A). FA and *errα* siRNA also interacted to affect *fas*, *acca* and *pparγ* mRNA amounts (Fig. 7B). Compared with the control, FA incubation increased the mRNA levels of *fas*, *acca* and *pparγ* and *errα* knockdown alleviated the FA-induced increment of *fas*, *acca* and *pparγ* mRNA abundances (Fig. 7B). The JASPAR predictions implied the potential binding sites of Errα with in the promoters of *fas*, *acca* and *pparγ* (Fig. 7C). For the *fas* promoter, we predicted Errα responsive element 1 (ERRE1) and 2 (ERRE2) at −1,667 to −1,655 bp and −1,782 to −1,770 of *fas* promoter, respectively. Compared with control, Errα overexpression increased the activities of *fas* promoter, but the ERRE1 mutation attenuated the increment of *fas* promoter activity induced by Errα overexpression. Meanwhile, the ERRE2 mutation vector did not affect the increment of activity of the *fas* promoter induced by Errα overexpression. The mutation vector of ERRE1 with ERRE2 abrogated Errα overexpression-induced increment of *fas* promoter activity, and the mutation vector of ERRE1 with ERRE2 showed no difference in *fas* promoter activity with the ERRE1 mutation vector (Fig. 7D). For *acca* and *pparγ* promoters, we predicted the Errα responsive element (ERRE) at −1,290 to −1,278 bp of *acca* promoter and at −250 to −240 bp of *pparγ* promoter, respectively. Compared with the control, Errα overexpression increased the activities of *acca* and *pparγ* promoters. The ERRE mutation attenuated the increment of *acca* and *pparγ* promoter activities induced by Errα overexpression (Fig. 7E and F). The EMSA showed that the putative Errα binding sites of *fas*, *acca* and *pparγ* promoters could bind with the nuclear extract directly, respectively. However, the binding can be disrupted by the unlabeled WT probe but restored by the mutant probe (Fig. 7G). Overall, these results support that ERRα targets *fas*, *acca* and *pparγ* genes, whose activation contributed FA-induced lipid accumulation.

## Discussion

Lipids are important energy-supplying substances for the vertebrates. High dietary lipid intake easily induces excessive body lipid accumulation [1, 2]. However, the mechanism remains unknown. Our present innovative findings included: (1) HFD upregulated hepatic lipogenic metabolism and lipotoxicity; (2) HFD facilitated hepatic mitochondrial fragmentation and damaged mitochondrial function; (3) HFD inhibited mitophagy, reduced Errα ubiquitination and increased Errα protein expression; (4) HFD-induced mitophagy and Errα activation are Parkin-dependent; (5) Parkin ubiquitinated Errα, and K63 was an important ubiquitination site for deubiquitinating Parkin activity; (6) ERRα targeted *fas*, *acca* and *pparγ* genes, whose activation contributed to FA-induced lipogenic metabolism and lipid accumulation. Overall, our findings demonstrate the HFD induced hepatic lipogenesis and lipotoxicity via Parkin-dependent mitophagy and Errα signal, and accordingly mitophagy and Errα are two important targets for the treatment against HFD-induced lipotoxicity and NAFLD.

Hepatic lipid accumulation is regulated by a complex network, including lipid uptake, de novo lipogenesis, and cholesterol synthesis [3]. Similar with previous studies [28–30], H&E, Oil Red O staining and qPCR analysis were used to determine hepatic lipid levels. In the present study, dietary fat addition induced the TG accumulation, and upregulated lipogenesis (*fas*, *acca*, *srebp1c*, *pparγ*). Taken together, high dietary fat addition increased lipid accumulation in yellow catfish liver.

Mitochondria are key intracellular energy-supplying organelle, and dysfunctional mitochondria are often related to the NAFLD pathogenesis [2]. In the present study, dietary fat addition induced mitochondrial swelling and damage, and decreased the mitochondrial copy numbers, in agreement with recent study [36]. Mitochondria is a highly dynamic organelles, and its homeostasis was maintained via specific fusion and fission proteins [37, 38]. In the present study, high dietary fat up-regulated the mRNA abundances of mitochondrial fission (*drp1*, *fis1*, *mief1* and *mief2*) and the protein level of mitochondrial fission (Drp1), but decreased the mRNA and protein levels of mitochondrial fusion (Opa1, Mfn2). Similarly, recent studies pointed out that lipid overload increased the Drp1 protein expression in the rat heart [38]. Chen et al. pointed out that HFD reduced the expression of MFN1, MFN2 and OPA1 (mitochondrial fusion proteins) in the myocardia [40]. Thus, dietary fat addition and FA incubation damaged mitochondria function. ATP was produced via mitochondrial respiration [5], and ATP depletion was considered the major marker of mitochondrial dysfunction [39, 40]. In the present study, dietary fat addition and FA incubation decreased ATP content. Taken together, our data indicated that high dietary fat addition disrupted the fusion-fission balance, and damaged mitochondrial function in liver of yellow catfish.

Excess lipids caused damaged mitochondria. In response to mitochondrial damage, mitophagy selectively eliminated damaged mitochondria [5]. However, the role of mitophagy in HFD-induced lipid accumulation requires further investigation. Other studies suggested excessive dietary uptake of saturated fatty acids (SFAs) causes mitochondrial dysfunction and metabolic disorders, while unsaturated fatty acids (UFAs) counterbalance these detrimental effects [41]. In this study, the percentage of SFA in the feed increased most significantly with increasing lipid levels. Thus, SFA is a potential functional fatty acid for the inhibition of mitophagy by HFD in this study. Furthermore, in present study, HFD reduced mRNA abundances of Ub-dependent mitophagy-related genes (*pink1* and *parkin*), but did not affect mRNA abundances of Ub-independent mitophagy-related genes (*fundc1*, *bnip3*, *bcl2l13* and *phb2*). Parkin-dependent mitophagy was induced by protein kinase PINK1 and parkin RBR E3 ubiquitin protein ligase with mitophagy receptors such as NDP52 and OPTN [7]. Ub-independent mitophagy preferentially interacted with GABARAP and LC3 proteins to recruit the dysfunctional mitochondria into the autophagosomes [5]. In the present study, high dietary fat addition and FA incubation reduced the Parkin protein level in the yellow catfish. Similarly, Li et al. pointed out that HFD impaired hepatic mitophagy and reduced Parkin protein expression in male [9].

In NALFD, mitophagy was inhibited and defective mitophagy is considered an important factor for NAFLD development [5, 10]. However, the potential role of mitophagy in HFD-induced lipid accumulation remains largely unknown. PARKIN is known to function in Parkin-dependent mitophagy, while FUNDC1 and BNIP3 contribute to Parkin-independent mitophagy [5]. Studies have suggested the beneficial function of mitophagy in preventing hepatic diseases. For example, Zhou et al. suggested that FUNDC1-mediated mitophagy suppressed hepatocarcinogenesis by inhibiting inflammasome response [15]. BNIP3 knockout mice increased lipid synthesis by increasing expression of lipogenic genes [42]. In the present study, high dietary fat addition and FA incubation escalated the mRNA and protein levels of Parkin-dependent mitophagy (Parkin, Lc3ii, P62, and Tom20), but did not affect Parkin-independent mitophagy (Fundc1, Bnip3, Bcl2l13 and Phb2). Similar results have been suggested in other studies [9, 10], but mechanism of mitophagy regulating lipid accumulation required further exploration. Notably, Parkin is an important key protein in initiating mitophagy [10], and Kim et al. pointed out that Parkin was a lipid-responsive regulator by modulating the CD36 ubiquitylation [16]. Thus, western blot were used to determine the protein levels of Parkin-dependent mitophagy (Parkin, Lc3ii, P62, and Tom20), based on previous studies [28, 29]. In present study, dietary fat addition inhibited the Parkin protein expression, in agreement with another report. Furthermore, *parkin*-knockdown further exacerbated lipid accumulation under FA incubation. In contrast, exercise promoted the increases of Parkin protein levels to maintain mitochondrial quality control in NAFLD patients [17]. Here, the NMN was used to activate the protein expression of Parkin, thereby activating Parkin-dependent mitophagy, and the activated mitophagy alleviates lipid accumulation under FA incubation. Taken together, our study innovatively found that the inhibition of Parkin contributed to lipid accumulation and the inhibition of mitophagy under FA incubation.

Errα plays a central role in controlling many metabolic processes such as fatty acid oxidation, lipogenesis, cholesterol homeostasis [43]. Previous reports showed that ERRα mediated the regulation of HFD-induced fat accumulation [44], but the mechanisms require further exploration. In present study, dietary fat addition and FA incubation increased the Errα protein levels. Audet-Walsh and Giguére pointed out that ERRα was one of Parkin target genes, but the relevant mechanism was unclear [18]. For the first time, immunoprecipitation (IP) was used to test the interaction between Parkin and Errα by using HEK293 T cell. In the present study, the ubiquitination of Errα and the interaction between Parkin and Errα were inhibited by FA incubation, and the deubiquitinating activity of Parkin was significantly related to the ubiquitination site K63. Over all, our study suggested that Errα was a new target gene of Parkin under dietary fat addition and FA incubation, and K63 was an important ubiquitination site for deubiquitinating Parkin activity.

Moreover, we predicted that the putative Errα binding sites in *fas*, *acca* and *pparγ* promoters, respectively. The *fas*, *acca* and *pparγ* are essential lipogenic genes [45, 46], but their relationship with Errα requires further exploration. In the present study, *Errα*-knockdown reversed the increase of mRNA abundances of lipogenic genes (*fas*, *acca* and *pparγ*) and the induction of TG content under FA incubation, in agreement with the report in mice [47]. Similarly with other studies [29, 31, 32], Luciferase activity measurement and electrophoretic mobility shift assay were used to determine the direct binding sites of promoter regions. Our further investigation showed that the ERRα bound to the ERRE on the *fas*, *acca* and *pparγ* promoters, respectively, which increased their transcriptional activities. Fas and Acca are important lipogenic enzymes [45], and Pparγ is a key transcription factor for controlling lipogenic metabolism [34]. Thus, we found that Errα directly bound to *fas*, *acca* and *pparγ* promoters, and increased *fas*, *acca* and *pparγ* transcription levels and activities, thereby contributing to HFD- and FA-induced lipid accumulation.

## Conclusion

**Fig. 8:**
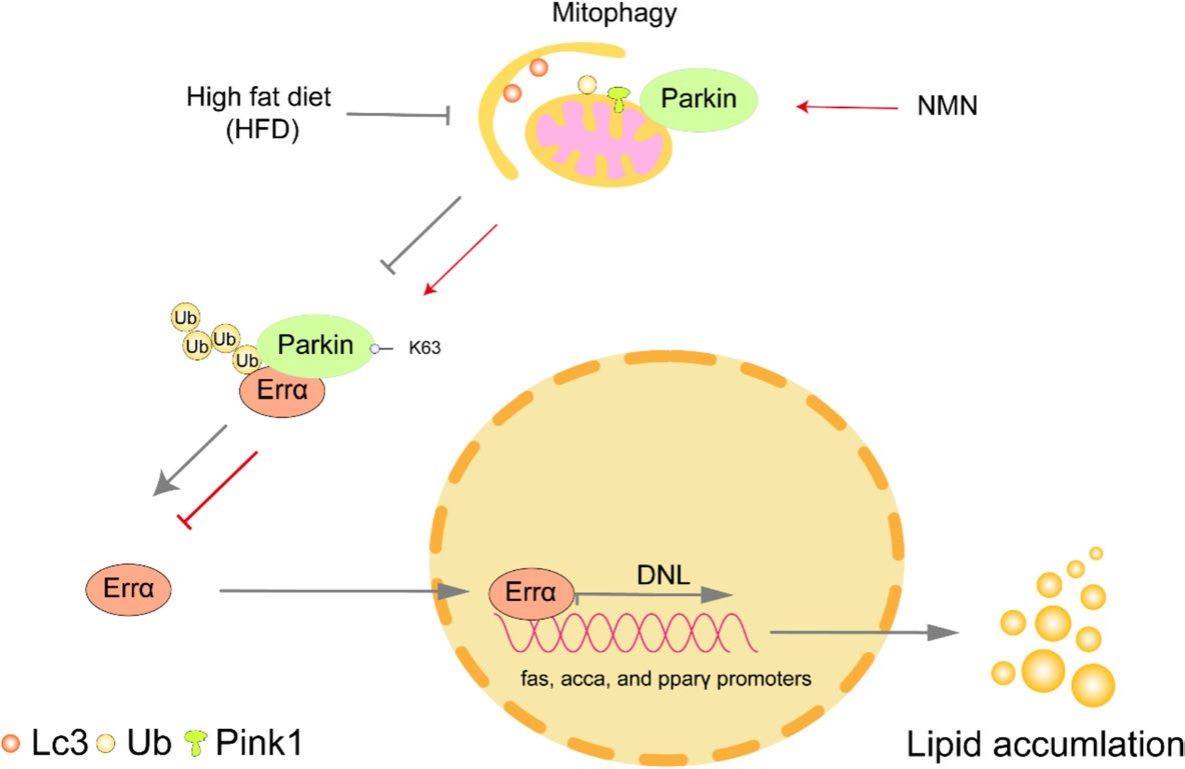
An innovative mechanism underlying high dietary fat-induced hepatic lipid accumulation. In this study, dietary fat addition inhibited Parkin-dependent mitophagy, damaged the mitochondrial function, reduced the Errα ubiquitination, and increased lipid accumulation. Parkin ubiquitinated Errα, and K63 was a key ubiquitination site for deubiquitinating Parkin activity. FA-induced inhibition of Errα ubiquitination promoted transcriptional activation of *fas*, *acca*, and *pparγ*, which contributed to high fat diet-induced lipogenesis and lipid accumulation

We propose an innovative mechanism underlying HFD-induced hepatic lipid accumulation (Fig. 8). High dietary fat intake induced lipid accumulation and inhibited mitophagy and disrupted the mitochondrial function. For the first time, we found that HFD reduced the ubiquitination of Errα and increased Errα protein expression by reducing Parkin protein expression, and K63 was an important ubiquitination site for deubiquitinating Parkin activity. Mechanistically, the inhibition of Parkin contributed to HFD-induced lipogenic metabolism and lipotoxicity via mitophagy and Errα signal. Overall, our study provided new targets against HFD-induced hepatic lipid accumulation and NAFLD in the vertebrates.

## Supplementary Information

Additional file 1: Text S1. Yellow catfish primary hepatocytes isolation and culture.

Additional file 2: Text S2. Vectors construction and mutation.

Additional file 3: Text S3. H&E and ORO staining.

Additional file 4: Text S4. Analysis of nutrient and fatty acid composition.

Additional file 5: Text S5. RNA isolation and real‑time quantitative PCR.

Additional file 6: Text S6. Western blot, Immunoprecipitation assays and Immunofluorescence staining.

Additional file 7: Table S1. Feed formulation and proximate analysis of experimental diets.

Additional file 8: Table S2. Effect of dietary lipid concentrations on growth performance and morphometrical parameters of yellow catfish.

Additional file 9: Table S3. Primers used for plasmid construction of si-*parkin* and si-*errα*.

Additional file 10: Table S4. Primers used for plasmid construction of Parkin and Errα into pcDNA3.1vector and site-mutation of Parkin.

Additional file 11: Table S5. Primers used for quantitative real-time PCR analysis.

Additional file 12: Table S6. Primers used for site-mutation analysis of *fas*, *acca* and *pparγ* promoters.

Additional file 13: Table S7. Primers used for electrophoretic mobility-shift assay.

Additional file 14: Table S8. Fatty acid compositions of the experimental diets, g/kg feed.

Additional file 15: Table S9. Fatty acid compositions of the experimental diets, % of total fatty acids.

Additional file 16: Fig. S1. Cell viability of primary hepatocytes under FA incubation.

Additional file 17: Fig. S2. TG content of primary hepatocytes under FA incubation.

Additional file 18: Fig. S3. Cell viability of primary IECs under NMN treatment.

Additional file 19: Fig. S4. A Relative mRNA expression of *parkin* after si‑*parkin* and si-*errα* knockdown. B Relative mRNA expression of *errα* after si-*errα* knockdown.

## Data Availability

All data generated or analyzed during this study are included in this published article.

## Abbreviations

ACCA: Acetyl CoA carboxylase
ATP: Adenosine triphosphate
BCL2L13: BCL2 like 13
BNIP3: BCL-2 19-kDa interacting protein 3
Drp1: Dynamin related protein 1
EMSA: Electrophoretic mobility shift assay
Errα: Estrogen-related receptor alpha
Fas: Fatty acid synthase
FUNDC1: FUN14 domain containing 1
H&E: Hematoxylin & eosin
LD: Lipid droplet
Mfn2: Mitofusin 2
Mief1: Mitochondrial elongation factor 1
Mief2: Mitochondrial elongation factor 2
NAFLD: Non-alcoholic fatty liver disease
NASH: Non-alcoholic steatohepatitis
Opa1: Optic atrophy 1
ORO: Oil Red O
Pink1: PTEN-induced putative kinase 1
Parkin: Parkin RBR E3 ubiquitin protein ligase
Ppar-γ: Peroxisome proliferator activated receptor γ
