# Supplementary material for: High fat diet (HFD) induced hepatic lipogenic metabolism and lipotoxicity via Parkin-dependent mitophagy and Errα signal of Pelteobagrus fulvidraco
Source: J Anim Sci Biotechnol. 2025 May 21;16:71. doi: 10.1186/s40104-025-01200-1 (PMC12093751; doi:10.1186/s40104-025-01200-1)
Supplement: Supplementary file 1 — Additional file 1: Text S1. Yellow catfish primary hepatocytes isolation and culture. [file 40104_2025_1200_MOESM1_ESM.docx]

**Additional file 1: Text S1**

**Yellow catfish primary hepatocytes isolation and culture**

Firstly, yellow catfish was sanitized with 75% alcohol and cleared of blood. Then, the liver was excised and transferred onto a plastic petri dish, and washed with PBS supplemented with streptomycin (100 mg/mL) and penicillin (100 IU/mL). Thirdly, the liver tissue was minced into small pieces and digested with 0.25% sterile trypsin at room temperature for 30 min. Finally, the primary hepatocytes were collected and cultured in DMEM medium containing 1 mmol/L L-glutamine, 10% (v/v) FBS, penicillin (100 IU/mL) and streptomycin (100 mg/mL), in 5% CO_2_ at 28 °C. Notably, cells were counted based on the trypan blue exclusion method, and only those cultures with more than 95% cell viability were used for the in vitro experiments.
