# Supplementary material for: High fat diet (HFD) induced hepatic lipogenic metabolism and lipotoxicity via Parkin-dependent mitophagy and Errα signal of Pelteobagrus fulvidraco
Source: J Anim Sci Biotechnol. 2025 May 21;16:71. doi: 10.1186/s40104-025-01200-1 (PMC12093751; doi:10.1186/s40104-025-01200-1)
Supplement: Supplementary file 2 — Additional file 2: Text S2. Vectors construction and mutation. [file 40104_2025_1200_MOESM2_ESM.docx]

**Additional file 2: Text S2**

**Vectors construction and mutation**

The open reading frames (ORFs) of Parkin and Errα sequences were subcloned into the pcDNA3.1 (+) vector with HA-tag and Myc-tag, respectively, using ClonExpressTM Ⅱ One Step Cloning Kit (#C112, Vazyme, Piscataway, NJ, USA). The mutations of lysine residue K63 and threonine residue T240 were produced in the HA-Parkin plasmid by using the Mut Express II Fast Mutagenesis Kit (#C214, Vazyme).
