# Supplementary material for: High fat diet (HFD) induced hepatic lipogenic metabolism and lipotoxicity via Parkin-dependent mitophagy and Errα signal of Pelteobagrus fulvidraco
Source: J Anim Sci Biotechnol. 2025 May 21;16:71. doi: 10.1186/s40104-025-01200-1 (PMC12093751; doi:10.1186/s40104-025-01200-1)
Supplement: Supplementary file 3 — Additional file 3: Text S3. H&E and ORO staining. [file 40104_2025_1200_MOESM3_ESM.docx]

**Additional file 3: Text S3**

**H&E and ORO staining**

For hematoxylin and eosin (H&E) staining, liver samples were fixed in 4% buffered formalin for 24 h. Dehydrated in graded ethanol concentrations and embedded in paraffin wax, sagittal sections were stained with H&E, and then prepared for light microscopy. For ORO staining, liver tissues were frozen at −25 °C in a cryostat and sectioned at a 5-μm thickness. The frozen slides were air dried, fixed in cold 10% buffered formalin (120 min at 4 °C), rinsed in distilled water, and stained with the ORO solution (0.5 g oil red, 100 mL isopropanol, and 60 mL distilled water). Then, sections were rinsed for a few seconds in two changes of 60% isopropanol, and then prepared for light microscopy. The average grayscale intensity was recorded using Image-Pro Plus 6.0 software (NIH, Bethesda, MD, USA), and 10 fields from each sample were randomly examined and quantified by Image J.
