# Supplementary material for: High fat diet (HFD) induced hepatic lipogenic metabolism and lipotoxicity via Parkin-dependent mitophagy and Errα signal of Pelteobagrus fulvidraco
Source: J Anim Sci Biotechnol. 2025 May 21;16:71. doi: 10.1186/s40104-025-01200-1 (PMC12093751; doi:10.1186/s40104-025-01200-1)
Supplement: Supplementary file 4 — Additional file 4: Text S4. Analysis of nutrient and fatty acid composition. [file 40104_2025_1200_MOESM4_ESM.docx]

**Additional file 4: Text S4**

**Analysis of nutrient and fatty acid composition**

For standard methods, the moisture content was determined by drying the sample at 105 °C. The crude protein content was obtained by Kjeldahl method. Crude lipid content was measured by the Soxhlet ether extraction method. Ash content was obtained by heating at 550 °C muffle furnace for 8 h.

To analyze fatty acid composition, 0.2 g samples were homogenized in 0.8 mL chloroform/methanol (2:1, v/v) for total lipid extraction. The solution was centrifuged at 5,000 × *g* for 5 min after adding 300 μL ddH₂O, and discarded the supernatant. The residue was dried under nitrogen. Then, 1.5 mL boron trifluoride-methanol solution was added to dissolve the lipid, and then methyl esterification was performed for 4 h at 85 °C. 1.5 mL n-hexane was added, collected the upper-layer sample and dried under nitrogen. Finally, 50 μL n-hexane per mg of dry substance was added and analyzed by a gas-liquid chromatograph (Shimadzu GC-14 A, Japan) with a flame ionization detector and a Shimadzu C-R6A Chromato-Integrator.
