# Supplementary material for: High fat diet (HFD) induced hepatic lipogenic metabolism and lipotoxicity via Parkin-dependent mitophagy and Errα signal of Pelteobagrus fulvidraco
Source: J Anim Sci Biotechnol. 2025 May 21;16:71. doi: 10.1186/s40104-025-01200-1 (PMC12093751; doi:10.1186/s40104-025-01200-1)
Supplement: Supplementary file 5 — Additional file 5: Text S5. RNA isolation and real‑time quantitative PCR. [file 40104_2025_1200_MOESM5_ESM.docx]

**Additional file 5: Text S5**

**RNA isolation and real‑time quantitative PCR (qPCR)**

Total RNA concentration was determined using a NanoDrop spectrophotometer (Thermo Scientific, Waltham, MA, USA). One microgram of RNA was reverse transcribed to cDNA using a GoScript Reverse Transcription System (Promega, A5004). qPCR was performed using the ABI7500 real-time PCR detection system (ABI System) and SYBR^®^ Green PCR Master Mixes (Applied Biosystems) with specific primers. The thermal cycling condition was at 50 °C for 2 min, at 95 °C for 10 min; at 95 °C for 10 s, at 60 °C for 30 s, at 72 °C for 15 s with 35 cycles, at 55 °C for 15 s, at 95 °C for 15 s.
