# Supplementary material for: High fat diet (HFD) induced hepatic lipogenic metabolism and lipotoxicity via Parkin-dependent mitophagy and Errα signal of Pelteobagrus fulvidraco
Source: J Anim Sci Biotechnol. 2025 May 21;16:71. doi: 10.1186/s40104-025-01200-1 (PMC12093751; doi:10.1186/s40104-025-01200-1)
Supplement: Supplementary file 6 — Additional file 6: Text S6. Western blot, Immunoprecipitation assays and Immunofluorescence staining. [file 40104_2025_1200_MOESM6_ESM.docx]

**Additional file 6: Text S6**

**Western blot, immunoprecipitation assays and immunofluorescence staining**

For the Western blot, liver samples and cell lysates were homogenized in radio immunoprecipitation assay buffer (Thermo Fisher Scientific, USA) containing a protease inhibitor (Sigma-Aldrich). The protein concentrations were determined via a BCA protein assay kit (Beyotime, China). Protein aliquots (15 μg protein/lane) were separated on 10%, 12% or 15% SDS–polyacrylamide gel (depending on the molecular weight of the proteins to be tested), and then transferred to poly-vinylidene fluoride membranes (Millipore, USA). Next, membranes were blocked with 8% nonfat milk in 1×TBS-T (Tris-buffered saline with Tween) and incubated with primary antibodies at 4 °C overnight. Membranes were incubated with HRP-conjugated secondary antibody (Bio-Rad) at room temperature for 2 h. The bands were identified by the Odyssey Infrared Fluorescent Western Blots Imaging System (Li-Cor Bioscience) with the enhanced ECL substrate (Bio-Rad). Protein expression were quantified using Image Lab software (version 2.0.0, USA) and normalised to the levels of β-actin. Specific primary antibodies were listed below: anti-USP30 (ab219969, Abcam), anti-PINK1 (23274-1-AP, Proteintech, China), anti-PARKIN (14060-1-AP, Proteintech, China), anti-SQSTM1/P62 (ab56416, Abcam), anti-LC3B (18725-1-AP, Proteintech, China), anti-TOM20 (ab186735, Abcam), anti-DRP1 (A2586, ABclonal Technology), anti-OPA1 (A9833, ABclonal Technology), anti-MFF (A4874, ABclonal Technology), anti-MFN2 (ab205236, Abcam), anti-NDUFB8 (14794-1-AP, Proteintech), anti-SDHB (YT5450, ImmunoWay), anti-ATP5A1 (14676-1-AP, Proteintech), anti-ERRα (A1798, Abclonal), anti-NFκb (abab32536, Abcam), anti-Ubiquitin (A19686, Abclonal), anti-β-actin (AC006, Abclonal), anti-HA tag (ab236632, Abcam), and anti-Myc tag (ab32, Abcam).

For the Immunoprecipitation assays, the HA-Parkin vectors and the Myc-Errα vectors were co-transfected into the 293T cells, and the empty vectors were co-transfected as the control. After 24 h, the cells were incubated with anti-HA or anti-Myc antibodies and rotated overnight at 4 °C, followed by incubation with protein A/G -agarose beads (Santa Cruz Biotechnology) for 4 h. Subsequently, the complex was pelleted and washed with TBS-T buffer five times. Finally, Western blots were conducted to detect anti-HA tag and anti-Myc tag antibodies, respectively.

For the immunofluorescence staining, cells were fixed in 4% paraformaldehyde solution at room temperature for 20 min, washed twice in ice-cold PBS, permeabilized in PBS-Triton and incubated with the specific primary antibody mentioned above overnight at 4 °C. Next, cells were washed in ice-cold PBS, and incubated with the secondary antibody for 1 h. DAPI (Invitrogen) was used to stain the nucleus of hepatocytes. Images were acquired with the laser scanning confocal microscope (Leica, Wetzlar, Germany), and quantified by the Image J software.
