# Supplementary material for: High fat diet (HFD) induced hepatic lipogenic metabolism and lipotoxicity via Parkin-dependent mitophagy and Errα signal of Pelteobagrus fulvidraco
Source: J Anim Sci Biotechnol. 2025 May 21;16:71. doi: 10.1186/s40104-025-01200-1 (PMC12093751; doi:10.1186/s40104-025-01200-1)
Supplement: Supplementary file 7 — Additional file 7: Table S1. Feed formulation and proximate analysis of experimental diets. [file 40104_2025_1200_MOESM7_ESM.docx]

**Table S1** Feed formulation and proximate analysis of experimental diets

| **Ingredients, g/kg** | **Low fat diet** | **Middle fat diet** | **High fat diet** |
| --- | --- | --- | --- |
| Casein | 350 | 350 | 350 |
| Gelatin | 20 | 20 | 20 |
| Fish meal | 100 | 100 | 100 |
| Wheat flour | 200 | 200 | 200 |
| Fish oil | 15 | 45 | 60 |
| Corn oil | 15 | 45 | 60 |
| Betaine | 10 | 10 | 10 |
| Ascorbyl-2-polyphosphate | 10 | 10 | 10 |
| Ca(H_2_PO_4_)_2_·H_2_O | 10 | 10 | 10 |
| Vitamin premix | 5 | 5 | 5 |
| Mineral premix | 5 | 5 | 5 |
| NaCl | 10 | 10 | 10 |
| Cellulose | 250 | 190 | 160 |
| Total | 1,000 | 1,000 | 1,000 |
| Proximate analysis, % dry matter | | | |
| Dry matter | 93.61 | 92.92 | 92.82 |
| Crude protein | 40.54 | 40.09 | 39.34 |
| Lipid | 6.31 | 12.03 | 15.32 |
| Ash | 2.19 | 2.51 | 2.51 |
| Gross energy, MJ/kg | 15.17 | 17.32 | 18.54 |

^1^Vitamin premix (mg or IU per kg diet): retinylacetate, 10,000 IU; cholecalciferol, 1,000 IU; all-rac-a-tocopheryl acetate, 30 IU; menadione nicotinamide bisulfite, 7 mg; thiamine hydrochloride, 6 mg; riboflavin, 3 mg; pyridoxine hydrochloride, 12 mg; D-calcium pantothenate, 30 mg; niacin, 50 mg; biotin, 1 mg; folic acid, 6 mg; cyanocobalamine, 0.03 mg

^2^Mineral mixture (mg/kg diet): Ca(H_2_PO_3_)_2_·H_2_O, 1,000; FeSO_4_·7H_2_O, 40; ZnSO_4_·7H_2_O, 40; MnSO_4_·H_2_O, 40; CuSO_4_·5H_2_O, 2; CaIO_3_·6H_2_O, 3; Na_2_SeO_3_, 0.05; CoSO_4_, 0.05
