# Supplementary material for: High fat diet (HFD) induced hepatic lipogenic metabolism and lipotoxicity via Parkin-dependent mitophagy and Errα signal of Pelteobagrus fulvidraco
Source: J Anim Sci Biotechnol. 2025 May 21;16:71. doi: 10.1186/s40104-025-01200-1 (PMC12093751; doi:10.1186/s40104-025-01200-1)
Supplement: Supplementary file 8 — Additional file 8: Table S2. Effect of dietary lipid concentrations on growth performance and morphometrical parameters of yellow catfish. [file 40104_2025_1200_MOESM8_ESM.docx]

**Table S2** Effect of dietary lipid concentrations on growth performance and morphometrical parameters of yellow catfish

| **Item** | **LFD** | **MFD** | **HFD** |
| --- | --- | --- | --- |
| Survival | 98.89±1.11 | 100.00±0.00 | 98.89±1.11 |
| IBW, g/fish | 2.33±0.03 | 2.33±0.03 | 2.33±0.02 |
| FBW, g/fish | 5.54±0.13^a^ | 6.45±0.10^b^ | 7.48±0.23^c^ |
| WG^2^, % | 136.28±7.17^a^ | 176.97±7.33^b^ | 220.93±13.05^c^ |
| SGR^3^, %/d | 1.55±0.027^a^ | 1.82±0.047^b^ | 2.08±0.024^c^ |
| FI^4^, g/fish | 3.69±0.02^a^ | 4.23±0.04^a^ | 5.39±0.04^b^ |
| HSI^5^, % | 1.48±0.05^a^ | 1.78±0.04^b^ | 1.91±0.07^b^ |
| CF^6^, % | 1.11±0.05^a^ | 1.42±0.12^b^ | 1.74±0.04^c^ |
| FE^7^ | 0.94±0.04 | 0.97±0.05 | 0.95±0.30 |

Values are mean ± SEM (*n* = 3 replicate tanks). FBW, WG, SGR, FI and FE: replicates of 28–30 fish; HSI and CF: replicates of 12 fish. IBW, Initial mean body weight; FBW, final mean body weight; WG, weight gain; SGR, specific growth rate; FI, feed intake. HSI, Hepatosomatic index. CF, condition factor; FE, Feed efficiency.

^1^ Survival= 100 × final fish number/initial fish number

^2^ WG = (FBW − IBW)/IBW ×100

^3^ SGR = 100 × [ln (FBW) − ln (IBW)]/d

^4^ FI=dry feed fed (g)/fish numbers

^5^ HSI = 100 × (liver weight/body weight)

^6^ CF = 100 × (body weight, g)/(body length, cm)^3^

^7^ FE = wet weight gain (g)/dry feed fed (g)

^a–c^ In the same row, values without same superscript letter differ significant, as determined by one-way ANOVA, and further post hoc Duncan’s multiple range testing (*P* ≤ 0.05)
