# Supplementary material for: High fat diet (HFD) induced hepatic lipogenic metabolism and lipotoxicity via Parkin-dependent mitophagy and Errα signal of Pelteobagrus fulvidraco
Source: J Anim Sci Biotechnol. 2025 May 21;16:71. doi: 10.1186/s40104-025-01200-1 (PMC12093751; doi:10.1186/s40104-025-01200-1)
Supplement: Supplementary file 9 — Additional file 9: Table S3. Primers used for plasmid construction of si-parkin and si-errα. [file 40104_2025_1200_MOESM9_ESM.docx]

**Table S3** Primers used for plasmid construction of si-*parkin* and si-*errα*

| **Genes** | **Forward primer (5´→3´)** | **Reverse primer (5´→3´)** |
| --- | --- | --- |
| si-*parkin*-422 | GGCUGAGCAGAGGAGUAAATT | UUUACUCCUCUGCUCAGCCTT |
| si-*parkin*-681 | GCUUCUUCCUGAUAGGAUUTT | AAUCCUAUCAGGAAGAAGCTT |
| si-*parkin*-1140 | GGGUCUCUUUGUCGAGGAUTT | AUCCUCGACAAAGAGACCCTT |
| si-*errα*-185 | GCUACUCCUCACCCGUAUATT | UAUACGGGUGAGGAGUAGCTT |
| si-*errα*-796 | GCCAAACAUGUCCCAGGAUTT | AUCCUGGGACAUGUUUGGCTT |
| si-*errα*-1086 | GGUCUACAUAGAGGACAUUTT | AAUGUCCUCUAUGUAGACCTT |
