# Supplementary material for: High fat diet (HFD) induced hepatic lipogenic metabolism and lipotoxicity via Parkin-dependent mitophagy and Errα signal of Pelteobagrus fulvidraco
Source: J Anim Sci Biotechnol. 2025 May 21;16:71. doi: 10.1186/s40104-025-01200-1 (PMC12093751; doi:10.1186/s40104-025-01200-1)
Supplement: Supplementary file 10 — Additional file 10: Table S4. Primers used for plasmid construction of Parkin and Errα into pcDNA3.1vector and site-mutation of Parkin. [file 40104_2025_1200_MOESM10_ESM.docx]

**Table S4** Primers used for plasmid construction of Parkin and Errα into pcDNA3.1 (+) vector and site-mutation of Parkin

| **Genes** | **Forward primer (5´→3´)** | **Reverse primer (5´→3´)** |
| --- | --- | --- |
| Parkin | ctagcgtttaaacttaagcttATGATCGTGTATGTGCGCTTTAA | aacgggccctctagactcgagTCACAGATCCTCTTCAGAGATGAGTT |
| Errα | ctagcgtttaaacttaagcttATGTCTTCCAGAGAGCGCCG | aacgggccctctagactcgagCTACTTATCGTCGTCATCCTTGTAATC |
| Parkin-K63N | CGGGAaacCTACGTGTACGGTGCCGAACCTGC | TACACGTAGgttTCCCGGCTGAATGGATTTACA |
| Parkin-T240R | CTGTGTTcgaCGACTGAACGAGCGACAGTTCA | TCAGTCGtcgAACACAGTACATGTGGAAACAATCC |
