# Supplementary material for: High fat diet (HFD) induced hepatic lipogenic metabolism and lipotoxicity via Parkin-dependent mitophagy and Errα signal of Pelteobagrus fulvidraco
Source: J Anim Sci Biotechnol. 2025 May 21;16:71. doi: 10.1186/s40104-025-01200-1 (PMC12093751; doi:10.1186/s40104-025-01200-1)
Supplement: Supplementary file 11 — Additional file 11: Table S5. Primers used for quantitative real-time PCR analysis. [file 40104_2025_1200_MOESM11_ESM.docx]

**Table S5** Primers used for quantitative real-time PCR analysis

| **Genes** | **Forward primer (5´→3´)** | **Reverse primer (5´→3´)** | **Accession No.** |
| --- | --- | --- | --- |
| *fas* | AACTAAAGGCTGCTGGTTGCTA | CACCTTCCCGTCACAAACCTC | JN579124 |
| *acca* | GGGGTTTTCACGCTGCTTC | GGTTCTGATTGGGTCGTCCTG | JX992746 |
| *pparγ* | ACGCCCCGTTCGTTATCC | TGAGCAGAGTCACCTGGTCATTG | JX992741 |
| *srebp1c* | CTGGGTCATCGCTTCTTTGTG | TCCTTCGTTGGAGCTTTTGTCT | JX992742 |
| *atp8* | ACAATTAAACCCCGCCCCAT | GATTTAAGGGTCTCGGCGCT | YP004769575.1 |
| *eff1a* | ACAATTAAACCCCGCCCCAT | GATTTAAGGGTCTCGGCGCT | NC015888.1 |
| *­pink1* | TAAGTGTCAGGACGAACGGC | TCACCTCGGAACTTCACTGC | DW805567.1 |
| *parkin* | AGGAGGGAAAGTCAAACGGC | ACTCCTCTGCTCAGCCTACG | XM027150103.1 |
| *fundc1* | CGTGATTCGGTAACGCACTG | ACACCACCACTGATGTCGTC | XM027164723.1 |
| *bnip3* | CGCTCCAGCACCATGAGTAT | AAGATGGTGAGGTGGCGAAG | XM027163085.1 |
| *bcl2l13* | GTCACAGGGAAGGGCAAGTC | CTGAGAAGAGTCCGTGTGGC | XM027161842.1 |
| *phb2* | AGCCGACATTTTGCGTGAAT | CAGGGCTCCAGCACCAATAA | XM027158299.1 |
| *drp1* | AAAGACGAGTGAGGAAAACGGT | CTGCTGATGCCCTTGTTGGT | XM027135876.2 |
| *fis1* | ACCGTTTGTTTAGAGCATGAGG | CCAGCTCCTCCAATAGCACAA | XM027134462.1 |
| *mief1* | GGCTCTGGACCGAGATGATG | CGCTCAGCTAGTACCTCGTG | XM027142490.1 |
| *mief2* | CAGTCTGCAACTCAGAGCCA | TGTTGAGCTCGGGACACTTC | XM027143069.1 |
| *opa1* | ACACGTGGATCGTTCCTGAC | TGTAGTTTCACTGGACAGCAGG | XM027163410.1 |
| *mfn2* | TACTCCCCCTTTCGGTCGAT | CCCGTCTTTAAGGTGCGAGT | XM027166944.1 |
| *errα* | GCATGCTGTCAAGCTATAGTATTC | GCGCTCTCTGGAAGACATGA | XM047805600.1 |
| *β-actin* | GGACTCTGGTGATGGTGTGA | CTGTAGCCTCTCTCGGTCAG | EU161066 |
| *18s rRNA* | AGCTCGTAGTTGGATCTCGG | CGGGTATTCAGGCGAGTTTG | KP938527 |
| *ubce* | TCAAGAAGAGCCAGTGGAGG | TAGGGGTAGTCGATGGGGAA | KP938524 |
| *gapdh* | TTTCAGCGAGAGAGACCCAG | ATGACTCTCTTGGCACCTCC | KP938521 |
| *tuba* | TCAAAGCTGGAGTTCTCGGT | AATGGCCTCGTTATCCACCA | KP938526 |
| *b2m* | GCTGATCTGCCATGTGAGTG | TGTCTGACACTGCAGCTGTA | KP938520 |
| *rpl7* | GGCAAATGTACAGGAGCGAG | GCCTTGTTGAGCTTGACGAA | KP938522 |

**Abbreviations:** *fas*, fatty acid synthase; *accα,* acetyl CoA carboxylase; *srebp1c*, sterol regulatory element binding proteins-1c; *pparγ*, peroxisome proliferator-activated receptor γ; *atp8*, ATP synthase F0 subunit 8 (mitochondrion); *pink1*, PTEN induced kinase 1; *parkin*, parkin RBR E3 ubiquitin protein ligase; *fundc1*, FUN14 domain containing 1; *bnip3*, BCL2 interacting protein 3; *bcl2l13*, bcl2l13; *phb2*, prohibitin 2a; *drp1*, dynamin related protein 1; *fis1*, fission, mitochondrial 1; *mief1*, mitochondrial elongation factor 1; *mief2*, mitochondrial elongation factor 2; *opa1*, Optic Atrophy 1; *mfn2*, mitofusin 2; *errα*, estrogen-related receptor alpha; *β-actin*, actin, beta; *18S rRNA*, 18S ribosomal RNA; *ubce*, ubiquitin conjugating enzyme 9; *gapdh*, glyceraldehyde-3-phosphate dehydrogenase; *tubα*, alpha tubulin; *b2m*, beta-2-microglobulin; *rpl7*, ribosomal protein L7
