# Supplementary material for: High fat diet (HFD) induced hepatic lipogenic metabolism and lipotoxicity via Parkin-dependent mitophagy and Errα signal of Pelteobagrus fulvidraco
Source: J Anim Sci Biotechnol. 2025 May 21;16:71. doi: 10.1186/s40104-025-01200-1 (PMC12093751; doi:10.1186/s40104-025-01200-1)
Supplement: Supplementary file 12 — Additional file 12: Table S6. Primers used for site-mutation analysis of fas, acca and pparγ promoters. [file 40104_2025_1200_MOESM12_ESM.docx]

**Table S6** Primers used for site-mutation analysis of *fas*, *acca* and *pparγ* promoters

| **Gene** | **Primers** | **Forward primer (5'→3')** | **Reverse primer (5'→3')** |
| --- | --- | --- | --- |
| *fas* | ERRE1-FAS | agactgatcagtatgacttgATTTTTGGGTCACCTTGTATTTTG | agtcatactgatcagtcttaCAAAATACAAGGTGACCCAAAAAT |
|  | ERRE2-FAS | aaagcgcatgacatcggacgttTAAGGAAAATATTACATGAGTGAA | tccgatgtcatgcgctttTTAATTTATTCCGATTATCCA |
| *acca* | EERE-ACCA | ggatatatacatgagaccaTCTGACAAATGCTGGAAATGTAA | ggtctcatgtatatatccaTTACATTTCCAGCATTTGTCAGA |
| *pparγ* | PPRE-PPARγ | cttcgcatgtgctatgaatgAAATAAACGTGTGCACTGTGTT | ggcactataagtcatctctgtactcAACACAGTGCACACGTTTATTT |
