# Supplementary material for: High fat diet (HFD) induced hepatic lipogenic metabolism and lipotoxicity via Parkin-dependent mitophagy and Errα signal of Pelteobagrus fulvidraco
Source: J Anim Sci Biotechnol. 2025 May 21;16:71. doi: 10.1186/s40104-025-01200-1 (PMC12093751; doi:10.1186/s40104-025-01200-1)
Supplement: Supplementary file 13 — Additional file 13: Table S7. Primers used for electrophoretic mobility-shift assay. [file 40104_2025_1200_MOESM13_ESM.docx]

**Table S7** Primers used for electrophoretic mobility-shift assay

| **Primers** |  | **Forward primer (5'→3')** | **Reverse primer (5'→3')** |
| --- | --- | --- | --- |
| ERRE-FAS | Biotin-probe | TTACCAGTTCAAGTTCAGGGCTTGA | TCAAGCCCTGAATTGAACTGGTAA |
|  | Mutative-competitor | TTACCAGTAGTTCAAGTGGGCTTGA | TCAAGCCCACTTGAACTACTGGTAA |
| ERRE-ACCA | Biotin-probe | AAATAAACGTGTGCACTGTGTT | AACACAGTGCACACGTTTATTT |
|  | Mutative-competitor | AAAGCGCATGACATCGGACGTT | AACGTCCGATGTCATGCGCTTT |
| ERRE-PPARγ | Biotin-Probe | TCTGACAAATGCTGGAAATGTAA | TTACATTTCCAGCATTTGTCAGA |
|  | Mutative-competitor | TCCAGACGCGTACATGCGCTGAA | TTCAGCGCATGTACGCGTCTGGA |
