# Supplementary material for: High fat diet (HFD) induced hepatic lipogenic metabolism and lipotoxicity via Parkin-dependent mitophagy and Errα signal of Pelteobagrus fulvidraco
Source: J Anim Sci Biotechnol. 2025 May 21;16:71. doi: 10.1186/s40104-025-01200-1 (PMC12093751; doi:10.1186/s40104-025-01200-1)
Supplement: Supplementary file 14 — Additional file 14: Table S8. Fatty acid compositions of the experimental diets, g/kg feed. [file 40104_2025_1200_MOESM14_ESM.docx]

**Table S8** Fatty acid compositions of the experimental diets, g/kg feed

| Fatty acid | **LFD** | **MFD** | **HFD** |
| --- | --- | --- | --- |
| C14:0 | 0.63 | 1.02 | 1.15 |
| C16:0 | 2.64 | 4.55 | 5.58 |
| C18:0 | 0.41 | 0.56 | 0.68 |
| C20:0 | 0.11 | 0.32 | 0.42 |
| C22:0 | 0.13 | 0.42 | 0.56 |
| ∑SFA^1^ | 3.91 | 6.86 | 8.39 |
| C14:1 | 0.00 | 0.00 | 0.02 |
| C16:1 | 0.57 | 1.00 | 1.20 |
| C18:1 | 3.03 | 5.51 | 7.01 |
| C20:1n-11 | 0.02 | 0.05 | 0.21 |
| C22:1n-11 | 0.01 | 0.03 | 0.03 |
| ∑MUFA^2^ | 3.63 | 6.59 | 8.46 |
| C18:3n-3 | 0.17 | 0.32 | 0.28 |
| C18:4n-3 | 0.07 | 0.12 | 0.37 |
| C20:3n-3 | 0.06 | 0.15 | 0.07 |
| C20:4n-3 | 0.70 | 1.08 | 1.85 |
| C20:5n-3 | 0.33 | 0.56 | 0.02 |
| C22:5n-3 | 0.68 | 1.95 | 2.58 |
| C22:6n-3 | 0.04 | 0.05 | 0.07 |
| ∑n-3 PUFA^3^ | 2.05 | 4.24 | 5.24 |
| C18:2n-6 | 2.94 | 6.27 | 8.37 |
| C18:3n-6 | 0.06 | 0.07 | 0.04 |
| C20:2n-6 | 0.01 | 0.01 | 0.02 |
| C20:4n-6 | 0.01 | 0.02 | 0.13 |
| ∑n-6 PUFA^4^ | 3.03 | 6.37 | 8.55 |

LFD, low fat diet; MFD, middle fat diet; HFD, high fat diet

^1^SFA: saturated fatty acids

^2^MUFA: mono-unsaturated fatty acids

^3^n-3 PUFA: n-3 poly-unsaturated fatty acids

^4^n-6 PUFA: n-6 poly-unsaturated fatty acids
