# Supplementary material for: High fat diet (HFD) induced hepatic lipogenic metabolism and lipotoxicity via Parkin-dependent mitophagy and Errα signal of Pelteobagrus fulvidraco
Source: J Anim Sci Biotechnol. 2025 May 21;16:71. doi: 10.1186/s40104-025-01200-1 (PMC12093751; doi:10.1186/s40104-025-01200-1)
Supplement: Supplementary file 15 — Additional file 15: Table S9. Fatty acid compositions of the experimental diets, % of total fatty acids. [file 40104_2025_1200_MOESM15_ESM.docx]

**Table S9** Fatty acid compositions of the experimental diets, % of total fatty acids

| Fatty acid | **LFD** | **MFD** | **HFD** |
| --- | --- | --- | --- |
| C14:0 | 3.76 | 4.25 | 5.01 |
| C16:0 | 18.21 | 18.90 | 20.93 |
| C18:0 | 2.22 | 2.32 | 3.22 |
| C20:0 | 1.36 | 1.32 | 0.84 |
| C22:0 | 1.81 | 1.74 | 1.03 |
| ∑SFA^1^ | 27.37 | 28.53 | 31.02 |
| C14:1 | 0.05 | 0.01 | 0.01 |
| C16:1 | 3.90 | 4.17 | 4.49 |
| C18:1 | 22.87 | 22.89 | 23.97 |
| C20:1n-11 | 0.69 | 0.21 | 0.19 |
| C22:1n-11 | 0.10 | 0.11 | 0.09 |
| ∑MUFA^2^ | 27.62 | 27.39 | 28.74 |
| C18:3n-3 | 0.91 | 1.34 | 1.35 |
| C18:4n-3 | 1.22 | 0.51 | 0.56 |
| C20:3n-3 | 0.24 | 0.62 | 0.50 |
| C20:4n-3 | 6.04 | 4.51 | 5.55 |
| C20:5n-3 | 0.06 | 2.33 | 2.58 |
| C22:5n-3 | 8.42 | 8.10 | 5.42 |
| C22:6n-3 | 0.22 | 0.21 | 0.30 |
| ∑n-3 PUFA^3^ | 17.09 | 17.61 | 16.25 |
| C18:2n-6 | 27.31 | 26.05 | 23.32 |
| C18:3n-6 | 0.13 | 0.28 | 0.51 |
| C20:2n-6 | 0.05 | 0.05 | 0.08 |
| C20:4n-6 | 0.42 | 0.09 | 0.07 |
| ∑n-6 PUFA^4^ | 27.92 | 26.47 | 23.98 |

LFD, low fat diet; MFD, middle fat diet; HFD, high fat diet

^1^SFA: saturated fatty acids

^2^MUFA: mono-unsaturated fatty acids

^3^n-3 PUFA: n-3 poly-unsaturated fatty acids

^4^n-6 PUFA: n-6 poly-unsaturated fatty acids
