# Supplementary material for: High fat diet (HFD) induced hepatic lipogenic metabolism and lipotoxicity via Parkin-dependent mitophagy and Errα signal of Pelteobagrus fulvidraco
Source: J Anim Sci Biotechnol. 2025 May 21;16:71. doi: 10.1186/s40104-025-01200-1 (PMC12093751; doi:10.1186/s40104-025-01200-1)
Supplement: Supplementary file 18 — Additional file 18: Fig. S3. Cell viability of primary IECs under NMN treatment. [file 40104_2025_1200_MOESM18_ESM.docx]

**Fig. S3** Cell viability of primary IECs under NMN treatment
