# Supplementary material for: High fat diet (HFD) induced hepatic lipogenic metabolism and lipotoxicity via Parkin-dependent mitophagy and Errα signal of Pelteobagrus fulvidraco
Source: J Anim Sci Biotechnol. 2025 May 21;16:71. doi: 10.1186/s40104-025-01200-1 (PMC12093751; doi:10.1186/s40104-025-01200-1)
Supplement: Supplementary file 19 — Additional file 19: Fig. S4. A Relative mRNA expression of parkin after si‑parkin and si-errα knockdown. B Relative mRNA expression of errα after si-errα knockdown. [file 40104_2025_1200_MOESM19_ESM.docx]

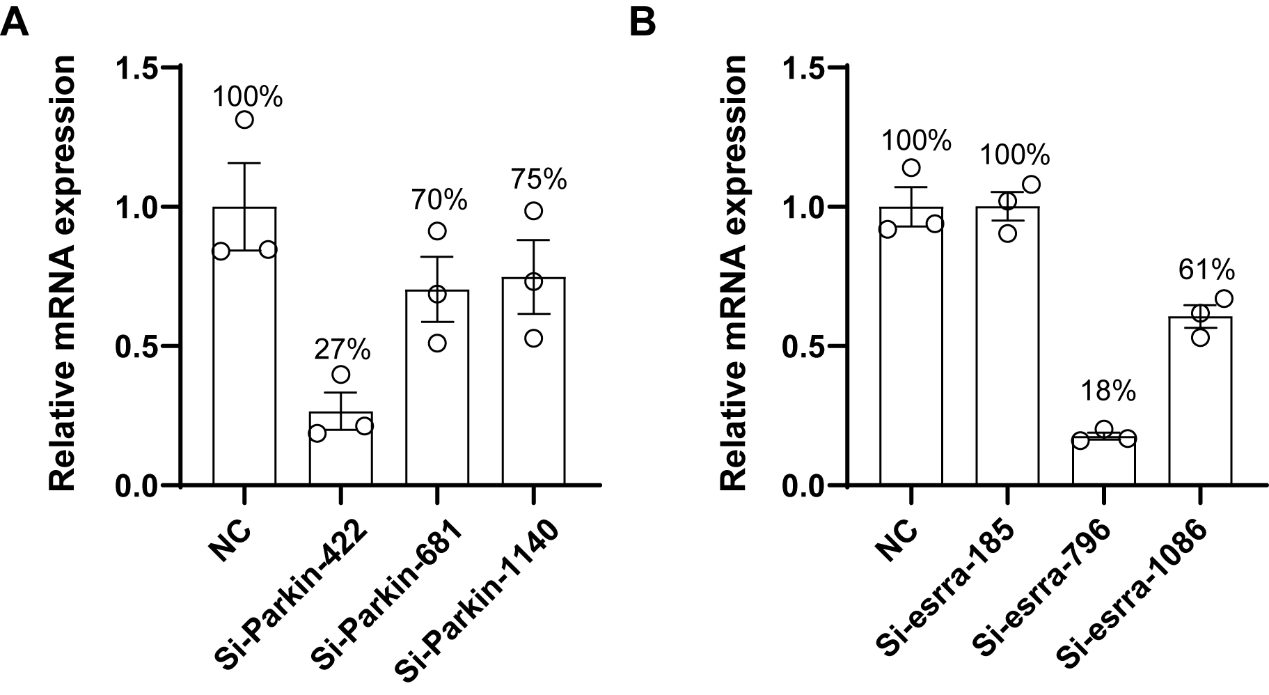
 **Fig. S4** **A** Relative mRNA expression of *parkin* after si‑*parkin* and si-*errα* knockdown*.* **B** Relative mRNA expression of *errα* after si-*errα* knockdown
